# Fractional Viscoelastic Modeling of Creep and Stress Relaxation Behaviors in Polymer-Based Energetic Materials

**DOI:** 10.3390/polym18121430

**Published:** 2026-06-08

**Authors:** Duo Gao, Wei Tang, Long Zhao, Hongwei Yuan

**Affiliations:** National Key Laboratory of Chemical Explosion Safety, Institute of Chemical Materials, China Academy of Engineering Physics, Mianyang 621900, China; gaoduo24@gscaep.ac.cn (D.G.); tangwei@caep.cn (W.T.); zhaolong@caep.cn (L.Z.)

**Keywords:** polymer-based energetic materials, fractional viscoelastic models, creep, stress relaxation, model comparison, extrapolation assessment

## Abstract

This study compares low-parameter fractional viscoelastic models for the unified characterization and extrapolation of creep and stress relaxation behaviors in polymer-based energetic materials, including polymer-bonded explosives (PBXs) and solid propellants. Fourteen candidate models composed of springs and spring-pot elements were considered under controlled parameter complexity. Their creep compliance and relaxation modulus were evaluated through Laplace-domain formulations, and the parameters were identified using a combined Talbot inverse Laplace transform and Gray Wolf Optimizer. Published creep and stress relaxation datasets were used to assess both fitting performance and early-stage data extrapolation behavior. The results show that the fractional Zener model and Model 13 can each describe both creep compliance and relaxation modulus within compact six-parameter rheological forms. Both models generally achieved coefficients of determination above 0.99. When the first 10% of the time span was used for calibration, the selected fractional models showed extrapolation capability over an approximately one-order-of-magnitude longer time window, with rRMSE values below 8.5% in reported cases and below 2% under suitable conditions. Compared with Prony series and power-law models, these fractional models offer compact alternatives for broad viscoelastic response characterization. These results provide guidance for selecting compact viscoelastic models for long-term response analysis of polymer-based energetic materials.

## 1. Introduction

Polymer-based energetic materials, including solid propellants and polymer-bonded explosives (PBXs), are extensively employed in modern military and aerospace applications owing to their superior formability and high energy density [1,2]. The polymeric binders in these composites impart pronounced viscoelastic characteristics, which govern their mechanical response under various loading conditions [3,4]. During long-term storage and transportation, structural components fabricated from such materials are subjected to sustained mechanical loading, resulting in time-dependent phenomena such as creep and stress relaxation [5]. These viscoelastic responses progressively degrade structural integrity and adversely affect detonation performance, while also posing potential safety hazards [6,7]. Consequently, accurate characterization and reliable extrapolation of the viscoelastic behavior of polymer-based energetic materials are of paramount importance for ensuring operational reliability and service life assessment.

Experimental investigations have demonstrated that polymer-based energetic materials frequently exhibit power-law behavior during creep and relaxation, and this behavior is not adequately captured by classical exponential-based models such as the Kelvin–Voigt or Maxwell models [8,9]. To capture these power-law behaviors, empirical models are frequently adopted in engineering practice. For instance, Zhao et al. employed a power-law model based on modified time-hardening theory to fit the creep master curve of PBX [10], and Feng et al. utilized a similar approach to describe the creep response of solid propellants [11]. Alternatively, such behavior can be approximated using generalized classical viscoelastic models composed of multiple rheological elements, which collectively represent a broad spectrum of discrete relaxation or retardation times [12]. For example, Deng et al. applied the generalized Maxwell model and the Burgers model to characterize relaxation and creep in HTPB propellants, respectively, and further proposed a modified Burgers model to improve the quality of fit for long-term creep behavior [5]. Lin et al. employed a six-element rheological model to describe the creep behavior of TATB-based PBX [13]. However, these approaches exhibit inherent limitations. Classical viscoelastic models typically require a large number of parameters to achieve satisfactory fitting accuracy, which complicates parameter identification and restricts their practical applicability. In contrast, empirical models can effectively capture observed trends with fewer parameters; however, they usually lack a direct rheological analog, which may limit their interpretability and extrapolation outside the calibrated time range. Moreover, empirical models are sometimes interpreted within a viscoplastic framework, further restricting their general applicability.

In recent years, fractional calculus has emerged as a powerful mathematical tool in viscoelastic theory, attracting considerable attention. Fractional calculus generalizes classical integer-order differentiation and integration to fractional orders and provides a natural mathematical framework for describing intermediate behavior between elasticity and viscosity [9]. Scott Blair’s early work suggested that conventional combinations of Hooke’s law and Newtonian viscosity were insufficient to represent observed viscoelastic responses, and introduced the concept of a “quasi-property” bridging elasticity and viscosity, which can be mathematically described using fractional derivatives [14,15]. Subsequent developments replaced integer-order derivatives in classical constitutive equations with fractional-order operators, thereby yielding fractional viscoelastic models that employ fractional rheological elements (spring-pots) in place of classical dashpots and enable more complex constitutive extensions [16,17,18,19,20].

Fractional viscoelastic models offer several advantages, including compact mathematical representation, favorable properties in the Laplace domain, and an intrinsic ability to capture long-term memory effects [9,15,16]. These models have been successfully applied across diverse material systems, including polymers, biomaterials, and geomaterials [9,21]. In the context of polymer-based energetic materials, Fang et al. developed a fractional model to describe the relaxation and storage modulus of solid propellants [22], while Zhang et al. proposed an improved fractional model for characterizing the low-frequency fatigue response of NEPE propellants under constant strain amplitude [23]. Although various fractional viscoelastic models have been proposed, existing studies have mainly focused on fitting specific datasets or developing increasingly complex rheological architectures. Less attention has been paid to the systematic assessment of low-parameter fractional viscoelastic models for polymer-based energetic materials under a consistent fitting and evaluation procedure. In particular, the relative performance of such models in describing both creep compliance and stress relaxation moduli across PBX and solid propellant datasets remains insufficiently clarified. In addition, most previous studies focused on either creep or relaxation behavior for a single material. Systematic assessment across different material systems and rigorous evaluation of long-term extrapolative capability remain insufficient, limiting their practical utility for reliability assessment and service life prediction.

Motivated by these considerations, this study aims to provide a comparative assessment of low-parameter fractional viscoelastic models for the creep and relaxation behavior of polymer-based energetic materials. The main contribution is not the development of a fundamentally new fractional rheological theory, but rather the systematic assessment of compact fractional models under a consistent identification and evaluation procedure. Fourteen candidate models composed of springs and spring-pot elements were considered under controlled parameter complexity. Their fitting accuracy, parameter economy, and extrapolation behavior were assessed using published creep and relaxation datasets of PBXs and solid propellants. A combination of the Talbot inverse Laplace transform and the Gray Wolf Optimizer was used for their parameter identification, particularly because several candidate models do not have analytical time-domain solutions. Through comparison with Prony series and power-law models, this study identifies compact fractional models that can simultaneously represent creep and relaxation responses and provide early-stage data extrapolation over an approximately one-order-of-magnitude longer time window within the considered datasets while maintaining a limited number of parameters.

The remainder of this study is organized as follows. Section 2 introduces the theoretical foundations of fractional calculus and fractional rheological elements. Section 3 presents the procedures for model construction and parameter identification. Section 4 systematically compares the descriptive performance of fractional and classical models for creep and relaxation behavior. Section 5 evaluates the long-term extrapolation behavior of the selected models from early-stage data. Finally, Section 6 summarizes the main conclusions.

## 2. Theoretical Foundations of Fractional Viscoelastic Models

Traditional rheological models for viscoelastic materials are typically constructed through mechanical analogies based on combinations of springs and dashpots, which represent elastic and viscous behavior, respectively. Fractional viscoelastic models extend this framework by incorporating fractional derivative theory into classical constitutive formulations. This section introduces the theoretical foundations of fractional viscoelastic models.

### 2.1. Theory of Fractional Derivatives

Due to its widespread application in fractional modeling and clear physical interpretability [19,21,24,25], the Caputo definition is adopted as the fractional derivative operator in this work.

For a sufficiently smooth function f(t) of t, the Caputo fractional derivative of order α∈[0, 1] is defined as [15]:

(1) Dtα0Cf(t)=1Γ(1−α)∫0t(t−s)−αf′(s)ds,

where $D_{t}^{\alpha }$ denotes the fractional derivative operator, Γ(x) represents the Gamma function of x, and α is the fractional order.

The Laplace transform of the Caputo fractional derivative of order α∈[0, 1] is given by [15]:

(2) L{Dtα0Cf(t)}(s)=sαF(s)−sα−1f(0).

Under zero initial conditions, i.e., f(0)=0, the term involving the initial value vanishes.

### 2.2. Fractional Rheological Elements

Early fractional viscoelastic models were formulated by replacing the integer-order derivative terms in the constitutive equations of classical rheological models with fractional-order derivatives. This approach gave rise to fractional rheological elements, which were based on mechanical analogies with classical viscoelastic models [17,18].

**Figure 1:**
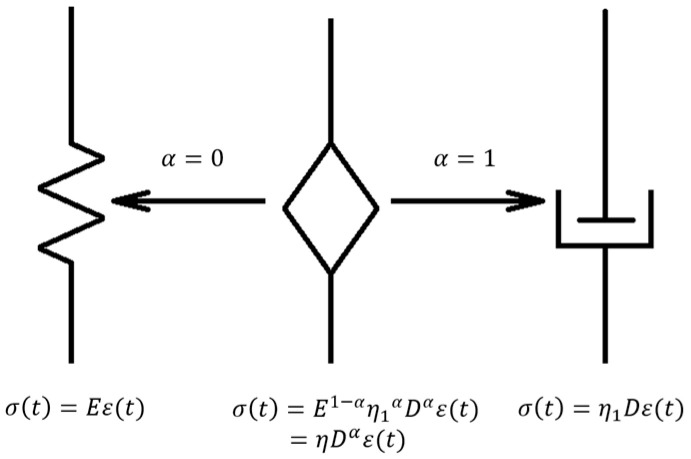
Schematic of the fractional rheological element (spring-pot), where E denotes the elastic modulus, η is the “firmness” and $\eta_{1}$ is the viscosity parameter. For the spring-pot relation $\sigma (t)=\eta D_{t}^{\alpha }\varepsilon (t)$, the parameter η has the unit of $MPa\cdot s^{\alpha }$ when strain is dimensionless. More generally, a spring-pot coefficient of order β has the unit of $MPa\cdot s^{\beta }$. These parameters should be interpreted as effective rheological coefficients rather than conventional viscosity constants.

Koeller referred to the fractional rheological element as a “spring-pot” and represented it by using a diamond-shaped symbol [16], as shown in Figure 1. This element is typically characterized by two parameters: η, which is often interpreted as the “firmness” of a material [26,27], and the fractional order α, with α∈[0,1]. When α=0, the element reduces to an elastic spring; when α=1, it reduces to a dashpot.

**Figure 2:**
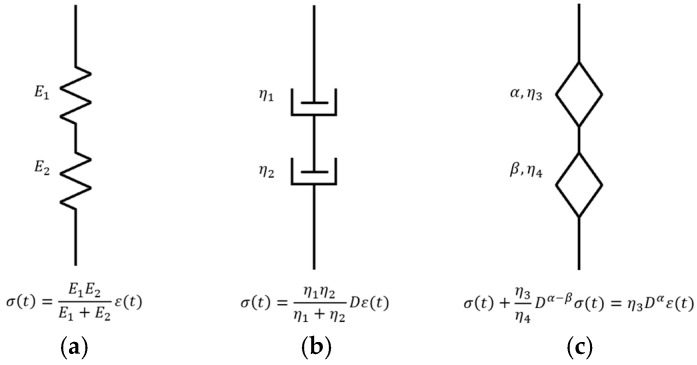
Mechanical analogy and constitutive equations of two elements in series: (**a**) spring; (**b**) dashpot; (**c**) fractional rheological element.

A notable distinction arises when considering combinations of individual rheological elements. For classical elements, combinations of springs alone or dashpots alone do not introduce additional time-dependent characteristics: springs remain purely elastic, while dashpots remain purely viscous, regardless of their series or parallel configurations. In contrast, fractional rheological elements inherently exhibit intermediate behavior between elasticity and viscosity. As illustrated in Figure 2, their combinations can generate richer time-dependent responses and introduce additional flexibility in representing viscoelastic behavior.

## 3. Construction of Fractional Viscoelastic Models for Polymer-Based Energetic Materials

This section describes the construction and calibration procedure for the candidate fractional viscoelastic models. First, the candidate model space and screening criteria are defined. Next, the Laplace-domain derivations of creep compliance and relaxation modulus are illustrated through a representative model. Finally, the parameter identification strategy based on numerical inverse Laplace transformation with a bio-inspired optimization algorithm is presented.

### 3.1. Fractional Viscoelastic Models for Polymer-Based Energetic Materials

In principle, fractional viscoelastic models can be assembled through arbitrary combinations of springs and spring-pots [15,19]. However, excessive complexity substantially increases computational cost and hinders parameter identifiability. In particular, Liu et al. referred to models containing three fractional rheological elements together with additional components as high-order fractional viscoelastic constitutive models [28]. The relaxation modulus expressions of their higher-order fractional models contain series terms and Mittag-Leffler functions with intricate parameters.

To balance descriptive capability, parameter efficiency, and engineering applicability, the candidate model space is restricted by two criteria: the number of rheological elements n≤4, and the number of model parameters m≤6.

These criteria were used to retain compact fractional viscoelastic models while excluding higher-order models that may introduce excessive parameter complexity, strong parameter coupling, reduced identifiability, and unstable long-term extrapolation. Degenerate configurations and models reducible to simpler equivalent forms were also excluded, yielding 14 representative fractional viscoelastic models for subsequent investigation. Their mechanical analogs, constitutive equations, and Laplace-domain expressions are summarized in Appendix A.

**Figure 3:**
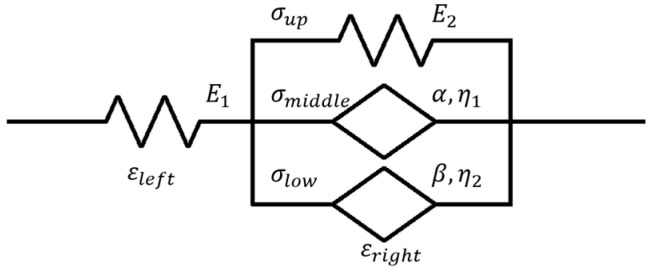
Mechanical analogy of Model 13 in Appendix A.

Because closed-form time-domain solutions are generally unavailable for these models, the Laplace-domain formulation coupled with numerical inversion is adopted as the principal solution strategy. To exemplify the derivation procedure, a six-parameter fractional model (Model 13) considered in this study is introduced below. As shown in Figure 3, this model comprises two classical springs ($E_{1}\;$, $E_{2}$) and two spring-pots ($\eta_{1},\;\alpha ;$ $\eta_{2},\beta$) arranged in a specific series-parallel configuration.

The left branch contains only the spring $E_{1}$, while the right branch consists of three parallel sub-branches: a spring $E_{2}$ and two spring-pots, ($\eta_{1},\;\alpha$) and ($\eta_{2},\beta$). Let $\varepsilon_{\mathrm{left}}(t)$ and $\varepsilon_{\mathrm{right}}(t)$ denote the strains in the left and right parts of the model, respectively, and let $\sigma_{\mathrm{up}}(t)$, $\sigma_{\mathrm{middle}}(t)$, and $\sigma_{\mathrm{low}}(t)$ represent the stresses in the upper, middle, and lower branches on the right, respectively. The kinematic and equilibrium relations hold as follows.

The total stress is equal to the sum of the stresses in the three branches on the right part:

(3) $$\sigma \left(t\right)=\sigma_{up}\left(t\right)+\sigma_{middle}\left(t\right)+\sigma_{low}\left(t\right).$$

The total strain is given by

(4) $$\varepsilon \left(t\right)=\varepsilon_{left}\left(t\right)+\varepsilon_{right}\left(t\right).$$

For the two springs, Hooke’s law gives

(5) $$\sigma (t)=E_{1}\varepsilon_{left}\left(t\right),$$

(6) $$\sigma_{up}\left(t\right)=E_{2}\varepsilon_{right}(t).$$

For the two fractional rheological elements, the constitutive relations are

(7) $$\sigma_{middle}\left(t\right)=\eta_{1}D^{\alpha }\varepsilon_{right}(t),$$

(8) $$\sigma_{low}\left(t\right)=\eta_{2}D^{\beta }\varepsilon_{right}(t).$$

Applying the Laplace transform to Equations (6)–(8) yields

(9) $$E_{right}(s)=\Sigma (s)\cdot \frac{1}{E_{2}+\eta_{1}s^{\alpha }+\eta_{2}s^{\beta }},$$

(10) $$E_{left}\left(s\right)=\Sigma \left(s\right)\cdot \frac{1}{E_{1}}.$$

Combining Equations (9) and (10), the Laplace-domain constitutive relation of Model 13 is obtained as

(11) $$E\left(s\right)=\Sigma \left(s\right)\cdot \frac{E_{1}+E_{2}+\eta_{1}s^{\alpha }+\eta_{2}s^{\beta }}{E_{1}E_{2}+E_{1}\eta_{1}s^{\alpha }+E_{1}\eta_{2}s^{\beta }},$$

where $E_{right}(s)$, $E_{left}\left(s\right),$ and Σ(s) denote the Laplace transforms of $\varepsilon_{right}\left(t\right)$, $\varepsilon_{left}\left(t\right),$ and σ(t), respectively. Taking the inverse Laplace transform of Equation (11) gives

(12) $$\sigma (t)+\frac{\eta_{1}}{E_{1}{+E}_{2}}D^{\alpha }\sigma (t)+\frac{\eta_{2}}{E_{1}{+E}_{2}}D^{\beta }\sigma (t)=\frac{E_{1}E_{2}}{E_{1}{+E}_{2}}\varepsilon (t)+\frac{E_{1}\eta_{1}}{E_{1}{+E}_{2}}D^{\alpha }\varepsilon (t)+\frac{E_{1}\eta_{2}}{E_{1}{+E}_{2}}D^{\beta }\varepsilon (t).$$

Equation (12) is the constitutive differential equation of Model 13. The same Laplace-domain procedure is applied to all 14 candidate models listed in Appendix A.

### 3.2. Expressions of Creep Compliance and Relaxation Modulus

Based on the Caputo fractional derivative, the Laplace-domain expressions for the creep compliance and relaxation modulus of Model 13 are derived using the Laplace transform.

Under zero initial conditions, all model variables and their derivatives vanish at $t\;=0^{-}$. For a step stress $\sigma_{0}H\left(t\right)$ applied at t = 0, the stress input can be written as

(13) $$\sigma \left(t\right)=\sigma_{0}H\left(t\right),$$

where H(t) denotes the Heaviside step function, and the Laplace transform of the stress input is

(14) $$\Sigma \left(s\right)=\sigma_{0}\cdot \frac{1}{s}.$$

Substituting Equations (13) and (14) into Equation (11) gives

(15) $$E\left(s\right)=\sigma_{0}\cdot \frac{1}{s}\cdot \frac{E_{1}+E_{2}+\eta_{1}s^{\alpha }+\eta_{2}s^{\beta }}{E_{1}E_{2}+E_{1}\eta_{1}s^{\alpha }+E_{1}\eta_{2}s^{\beta }},$$

from Equation (15), the Laplace-domain expression for the creep compliance J(s) is obtained as

(16) $$J\left(s\right)=\frac{Ε\left(s\right)}{\sigma_{0}}=\frac{E_{1}{+E}_{2}+\eta_{1}\cdot s^{\alpha }+\eta_{2}\cdot s^{\beta }}{E_{1}E_{2}\cdot s+E_{1}\eta_{1}\cdot s^{\alpha +1}+E_{1}\eta_{2}\cdot s^{\beta +1}}.$$

Similarly, under a step strain applied at t=0, the strain input and its Laplace transform can be written as

(17) $$\varepsilon \left(t\right)=\varepsilon_{0}H\left(t\right),$$

(18) $$Ε\left(s\right)=\varepsilon_{0}\cdot \frac{1}{s}.$$

Substituting Equations (17) and (18) into Equation (11) yields the Laplace-domain expression for the relaxation modulus G(s):

(19) $$G\left(s\right)=\frac{\Sigma \left(s\right)}{\varepsilon_{0}}=\frac{E_{1}E_{2}+E_{1}\eta_{1}\cdot s^{\alpha }+E_{1}\eta_{2}\cdot s^{\beta }}{{(E}_{1}{+E}_{2})\cdot s+\eta_{1}\cdot s^{\alpha +1}+\eta_{2}\cdot s^{\beta +1}}.$$

Following the procedure outlined above, the Laplace-domain expressions for the creep compliance and relaxation modulus of all 14 models listed in Appendix A were derived using the same approach.

### 3.3. Talbot–Gray Wolf Parameter Identification Procedure

Parameter identification for fractional viscoelastic models can be formulated as the minimization of the discrepancy between model responses and experimental data. Accordingly, the optimization problem considered here is defined as a bounded minimization problem for an error function. Since the number of data points is typically much larger than the number of model parameters, the error function is defined as the sum of the squared relative errors between model responses and experimental data [22]:

(20) $$Err=\sum_{i=1}^{n}{\left[\frac{f^{num}\left(\vec{x},t_{i}\right)-y_{i}}{y_{i}}\right]}^{2},$$

where n denotes the number of discrete data points, $\vec{x}=[x_{1},x_{2},\ldots ,x_{m}]$ is the model parameter vector, $t_{i}$ is the time at the i-th data point, $f^{\mathrm{num}}(\vec{x},t_{i})$ is the model response at time $t_{i}$ for the parameter vector $\vec{x}$, and $y_{i}$ represents the actual value at time $t_{i}$. The creep and relaxation datasets of polymer-based energetic materials are denoted uniformly by $y_{i}$. All model parameters are taken to be non-negative, and the fractional-order parameters are constrained to α∈[0,1], where the closed interval is adopted to include the degenerate cases of fractional rheological elements.

The expressions for creep compliance and relaxation modulus in many fractional viscoelastic models typically involve the Mittag-Leffler function, which makes gradient-based parameter identification difficult. Consequently, parameter identification for such models has attracted considerable attention. Various approaches, including Bayesian algorithms, interior-point methods, and Powell’s algorithm [22,29,30,31,32,33], have been employed in studies involving fractional calculus or Mittag-Leffler-type functions. However, closed-form time-domain expressions for most of the 14 models considered here are difficult to obtain, which further complicates parameter identification.

To address this issue, a parameter identification procedure combining the Talbot inverse Laplace method with the Gray Wolf Optimizer was adopted [34,35,36]. The numerical inverse Laplace method provides sufficient accuracy for engineering applications while reducing computational cost, and the Gray Wolf Optimizer is well-suited to gradient-free optimization problems. The overall optimization procedure is summarized in Algorithm 1.
**Algorithm 1.** Talbot–Gray Wolf parameter identification procedure1:Generate an initial population of gray wolves $X_{i}\;(i=1,\;2,\ldots ,\;N)$
2:Initialize the control parameters and termination condition3:**while** (termination condition is not met) **do**4:         **For** 
$\mathrm{each}\;\mathrm{wolf}\;X_{i}$ **do**
5:                   Compute the model response using the Talbot inverse Laplace method6:                   Evaluate the objective function $Err(X_{i})$ using the experimental data7:         
**end for**
8:          Rank the wolves according to 
$Err(X_{i})$
9:          Update the leading wolves  
$X_{\alpha }$
$,\;X_{\beta }$
$\;\mathrm{and}\;X_{\delta }$
10:          Update the positions of all wolves using the Gray Wolf Optimizer11:**end while**12:$\mathrm{return}\;X_{\alpha }$

The combined Talbot–Gray Wolf parameter identification procedure provides an effective strategy for parameter identification in the fractional viscoelastic models considered here. This framework was employed in all subsequent fitting and extrapolation analyses of fractional viscoelastic models. As shown in Table 1, to ensure reproducibility of the stochastic optimization, the random number generator was initialized with a fixed seed of 2025. The number of truncation terms in the Talbot inversion method was set to 64, and the Gray Wolf Optimizer was configured with a population size of 40 and a maximum of 500 iterations. The lower bounds of all parameters were set to 0, and the upper bounds of the fractional orders were fixed at 1, whereas those of the remaining parameters differed across datasets; the specific values are provided in the corresponding sections. Given the variations in material properties and loading conditions, no additional termination criteria were imposed in any of the subsequent parameter identification and calibration processes, so that the optimization was terminated solely upon reaching the maximum number of iterations.

The benchmark models, including the power-law model and the Prony series models, were identified using least-squares fitting with the same error definition as that used for the fractional models. For the Prony series models, the relaxation or retardation times were prescribed as uniformly spaced on a logarithmic time scale across the range, and the remaining coefficients were fitted accordingly. For the fitting comparison in Section 4, the coefficient of determination $R^{2}$ was used to quantify descriptive accuracy, while the Akaike information criterion (AIC) was introduced to account for differences in the number of fitted parameters. The AIC was calculated as AIC=nln(RSS/n)+2k, where *n* is the number of data points, *RSS* is the residual sum of squares, and *k* is the number of fitted parameters. A lower AIC indicates a better balance between fitting accuracy and model complexity. AIC values were compared only among models fitted to the same dataset. For the extrapolation assessment in Section 5, rRMSE was used to quantify the deviation between the extrapolated and experimental responses, with the calibration portion excluded from the calculation.

To further examine parameter identification stability and possible parameter coupling, 30 independent optimization runs with different random streams were performed for a representative fractional model and dataset. The resulting parameter distributions, correlation matrix, fitted curves, and extrapolated curves are provided in Appendix D.

## 4. Characterization of Fitting Performance of Fractional Viscoelastic Models

This section evaluates the descriptive performance of fractional viscoelastic models in comparison with classical models by using literature data on the creep and relaxation of PBX and solid propellants. Among the 14 candidate models, the fractional Zener model (Model 6 in Appendix A, denoted as “FZM” in the figures) and Model 13 consistently exhibited favorable descriptive performance across the datasets and are, therefore, highlighted here. For comparison, the power-law model and the Prony series forms of the generalized Maxwell or generalized Kelvin models are also included as benchmarks. The identified model parameters are provided in Appendix B. In the following comparisons, $R^{2}$, AIC, and residual plots are used together to evaluate fitting accuracy, parameter complexity, and error distribution.

### 4.1. Characterization of Relaxation Modulus

The Prony series representation is a standard tool in linear viscoelasticity. Its performance depends on the prescribed relaxation or retardation times, the number of terms, and the fitting or regularization strategy. Therefore, the present comparison should be interpreted under the adopted logarithmically spaced time-constant setting. For relaxation comparison, the empirical power-law model and the generalized Maxwell model in Prony series form are employed as benchmark models.

The Prony series expression for relaxation is given by

(21) $$G\left(t\right)=E_{\infty }+\sum_{i=1}^{n}E_{i}\exp\left(-\frac{t}{\tau_{i}}\right),$$

where $E_{\infty }$ is the equilibrium modulus, and $E_{i}$ represents the modulus of the i-th Maxwell branch; $\tau_{i}$ is the relaxation time of the i-th branch, and n is the number of Maxwell branches connected in parallel.

In addition, the empirical model for relaxation is given by

(22) $$G\left(t\right)=A+B\cdot t^{-\alpha },$$

where A, B, and α are parameters to be determined.

#### 4.1.1. Relaxation Modulus of Solid Propellant (PVC/AP)

**Figure 4:**
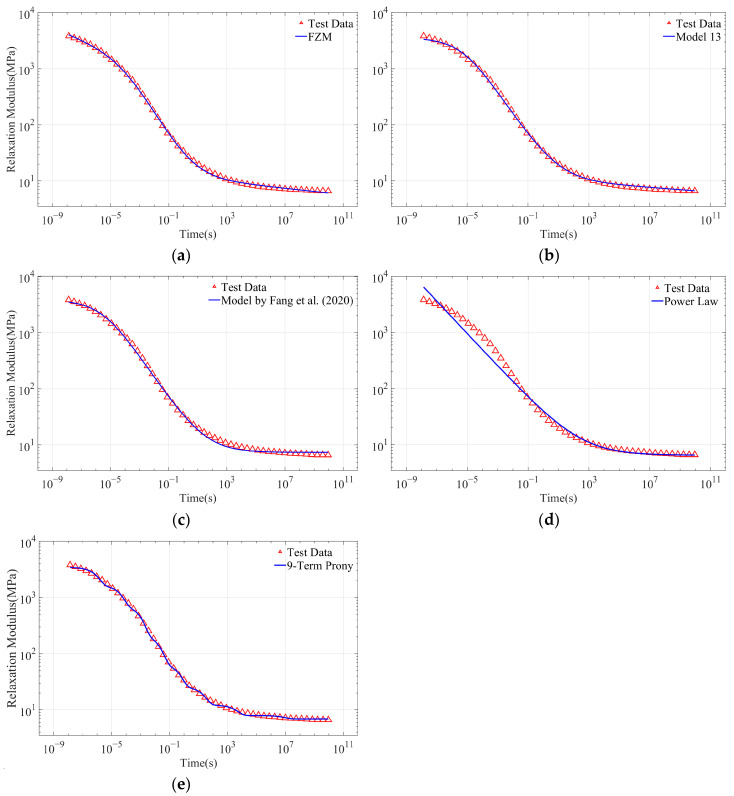
Fitting of relaxation data for solid propellant (PVC/AP) using fractional viscoelastic models and classical models: (**a**) fractional Zener model; (**b**) Model 13; (**c**) the fractional model proposed by Fang et al. [22]; (**d**) power-law model; (**e**) 9-term Prony series.

The relaxation master curve of a PVC/AP solid propellant was used to assess the models [37]. Fang et al. previously analyzed the same dataset using a five-element three-branch fractional Maxwell model and compared its performance with that of a 9-term Prony series [22]. The fractional Zener model, Model 13, the model of Fang et al., the power-law model, and the 9-term Prony series were evaluated comparatively. The upper bounds for all non-fractional-order parameters of fractional viscoelastic models were set to $10^{4}$. The corresponding fitting results are presented in Figure 4a–e and Table 2.

**Figure 5:**
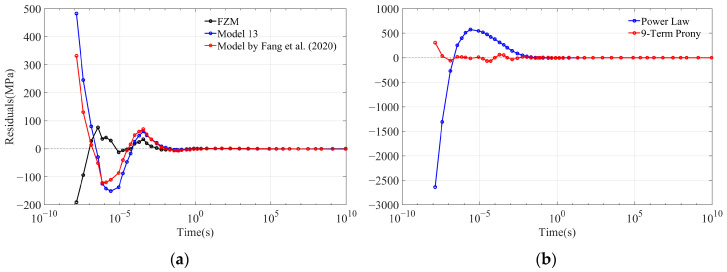
Residuals versus time of relaxation data for solid propellant (PVC/AP) using fractional viscoelastic models and classical models: (**a**) fractional Zener model, Model 13, and the fractional model proposed by Fang et al. [22]; (**b**) power-law model and 9-term Prony series.

As summarized in Table 2, the fractional Zener model, Model 13, Fang et al.’s fractional model [22], and the 9-term Prony series all achieved high fitting accuracy ($R^{2}>0.99$). The power-law model showed a much lower $R^{2}$ ($R^{2}=0.7772$), indicating that it was less suitable for this relaxation dataset. When both fitting accuracy and parameter complexity are considered, the fractional Zener model (m=6) gave the lowest AIC among all compared models, suggesting the most favorable balance between accuracy and parameter economy for this dataset. Although the 9-term Prony series also achieved a high $R^{2}$, it required a much larger number of parameters. Therefore, its complexity-penalized advantage was less pronounced than that of the fractional Zener model. Model 13 and Fang et al.’s fractional model [22] also provided accurate descriptions, but their AIC values were higher than that of the fractional Zener model in this case, which is consistent with the residual plots shown in Figure 5.

Given that the three-branch fractional Maxwell model falls outside the selection criteria of this study (n≤4, m≤6) and features a relatively complex expression for creep compliance, it was excluded from subsequent analyses.

#### 4.1.2. Relaxation Modulus of PBX

Xiao et al. reported the short-term relaxation master curve data ($10^{-4}-10^{4}\;s$) for PBX at 20 °C [38]. For comparison, the fitting results of the two fractional viscoelastic models, together with those of the power-law model and the Prony series, were presented for the PBX relaxation data, and the upper bounds for all non-fractional-order parameters of the fractional viscoelastic models were set to 3000.

**Figure 6:**
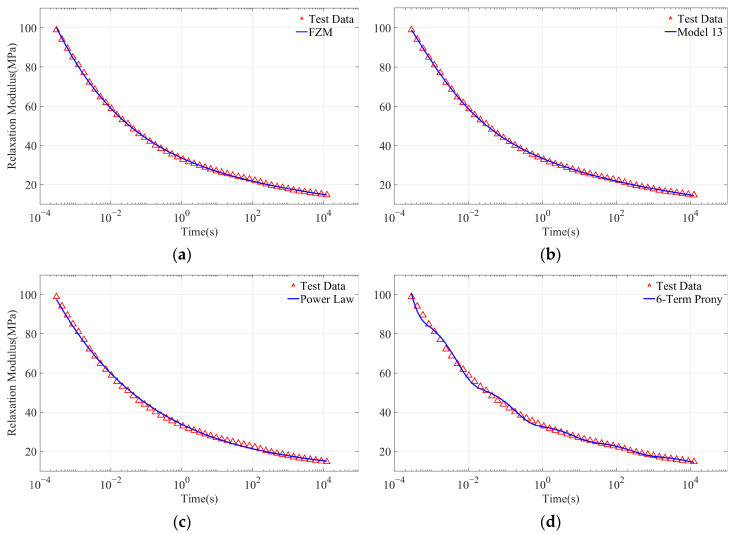
Fitting of relaxation data for PBX using fractional viscoelastic models and classical models: (**a**) fractional Zener model; (**b**) Model 13; (**c**) power-law model; (**d**) 6-term Prony series.

**Figure 7:**
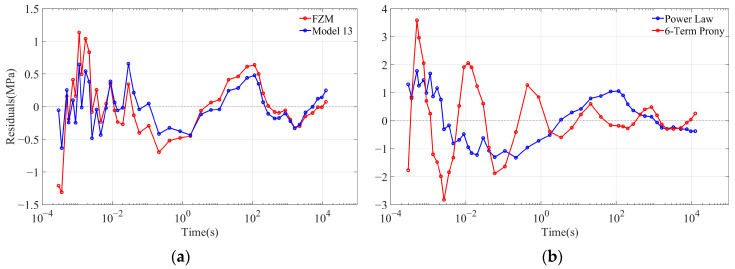
Residuals versus time of relaxation data for PBX using fractional viscoelastic models and classical models: (**a**) fractional Zener model and Model 13; (**b**) power-law model and 6-term Prony series.

Figure 6 illustrates the fitted curves together with the experimental data. The two fractional viscoelastic models, as well as the power-law model and the 6-term Prony series, all accurately described the short-term relaxation behavior of PBX, with $R^{2}\geq 0.9977$. However, the residual plots in Figure 7 and the AIC values in Table 3 further distinguish their complexity-penalized performance. Model 13 achieved the lowest AIC, followed by the fractional Zener model, indicating that the two fractional models provided a better balance between fitting accuracy and parameter complexity than the Prony series models under the present fitting protocol. Although the 6-term Prony series also achieved high $R^{2}$, its larger parameter number resulted in a less favorable AIC. These results support the use of compact fractional models for the short-term relaxation characterization of PBX.

### 4.2. Characterization of Creep Compliance

Consistent with the relaxation data, the creep data plotted on a logarithmic time scale were also obtained from the creep behavior of PBX and solid propellants. For comparison, the empirical power-law model for creep and the creep form of the generalized Kelvin model were employed. The expression of the Prony series for creep is given by

(23) $$J\left(t\right)=\frac{1}{J_{0}}+\sum_{i=1}^{n}\frac{1}{J_{i}}\left(1-\exp\left(-\frac{t}{\lambda_{i}}\right)\right),$$

where $\frac{1}{J_{0}}$ is the instantaneous compliance, and $\frac{1}{J_{i}}$ represents the compliance of the i-th Kelvin branch, $\lambda_{i}$ is the retardation time of the i-th branch, and n is the number of Kelvin branches connected in series. Meanwhile, the empirical model for creep is given by

(24) $$J\left(t\right)=A+B\cdot t^{\beta },$$

where A, B, and β are parameters to be determined.

#### 4.2.1. Creep Compliance of PBX

**Figure 8:**
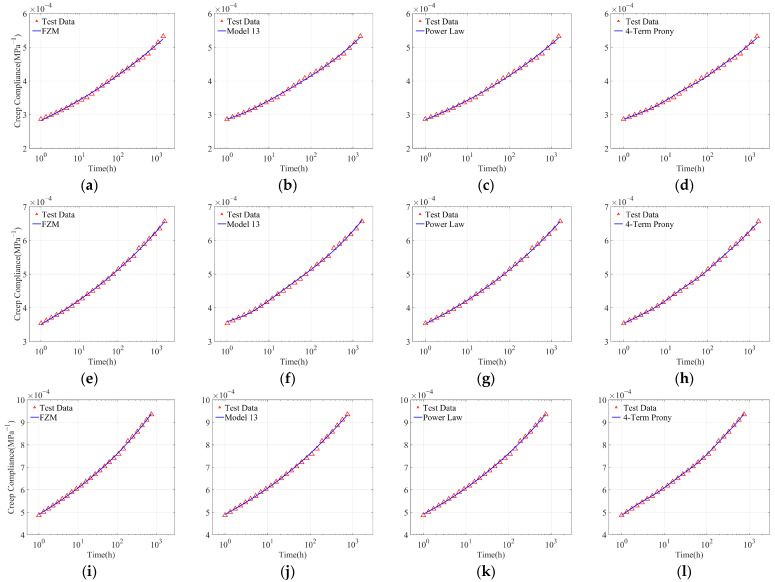
Fitting results for the compressive creep data of PBX under different load and temperature conditions: (**a**) fractional Zener model at −3 MPa and 40 °C; (**b**) Model 13 at −3 MPa and 40 °C; (**c**) power-law model at −3 MPa and 40 °C; (**d**) 4-term Prony series at −3 MPa and 40 °C; (**e**) fractional Zener model at −4 MPa and 40 °C; (**f**) Model 13 at −4 MPa and 40 °C; (**g**) power-law model at −4 MPa and 40 °C; (**h**) 4-term Prony series at −4 MPa and 40 °C; (**i**) fractional Zener model at −5 MPa and 50 °C; (**j**) Model 13 at −5 MPa and 50 °C; (**k**) power-law model at −5 MPa and 50 °C; (**l**) 4-term Prony series at −5 MPa and 50 °C.

**Figure 9:**
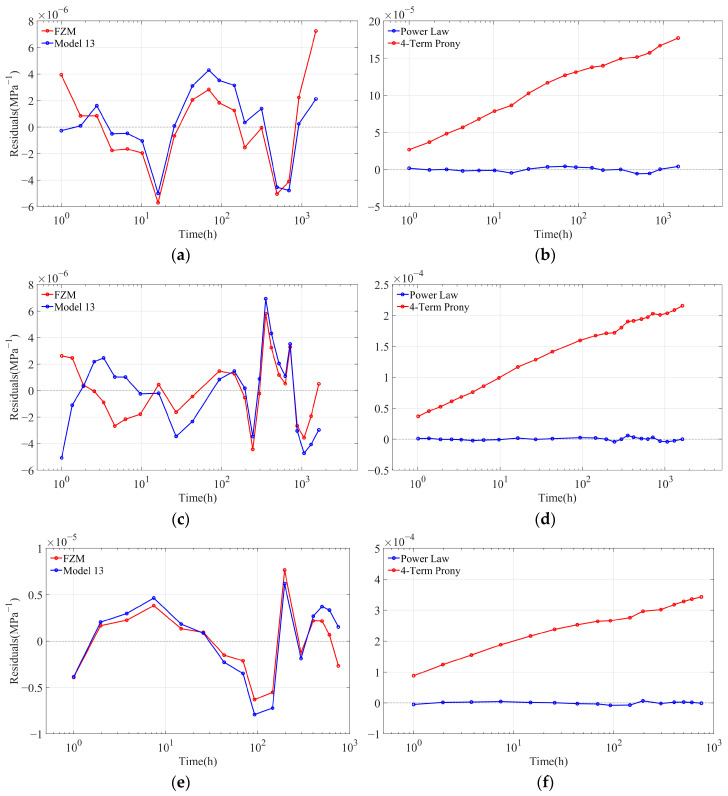
Residuals versus time for the fitted compressive creep data of PBX under different load and temperature conditions: (**a**) fractional Zener model and Model 13 at −3 MPa and 40 °C; (**b**) power-law model and 4-term Prony series at −3 MPa and 40 °C; (**c**) fractional Zener model and Model 13 at −4 MPa and 40 °C; (**d**) power-law model and 4-term Prony series at −4 MPa and 40 °C; (**e**) fractional Zener model and Model 13 at −5 MPa and 50 °C; (**f**) power-law model and 4-term Prony series at −5 MPa and 50 °C.

Thompson et al. [39] systematically investigated the creep behavior of PBX 9502 under various temperatures and tensile/compressive loads. Here, creep data under axial loads of −3 MPa (40 °C), −4 MPa (40 °C), and −5 MPa (50 °C) were employed for analysis. The data began at approximately 1 h; due to the absence of data in the glassy region ($t\;<\;10^{0}\;s$), 3-term and 4-term Prony-series models were employed for fitting. The fitting results and residual plots for each model are shown in Figure 8 and Figure 9, respectively. The upper bounds for all non-fractional-order parameters of fractional viscoelastic models were set to $10^{6}$. For compressive creep data, the creep compliance is reported as a positive magnitude, calculated from the absolute values of strain and compressive stress; the same convention was used consistently in fitting, AIC calculation, and error evaluation.

All compared models achieved satisfactory fitting performance for the compressive creep data of PBX, with $R^{2}$ values of at least 0.9971. As summarized in Table 4, the two fractional models accurately captured the creep behavior under all three loading conditions. However, when AIC is considered, the advantage of the fractional models becomes less uniform than that observed in the relaxation cases. The power-law model gave the lowest AIC at −3 MPa and −4 MPa, while the 4-term Prony series gave the lowest AIC at −5 MPa. This indicates that the PBX compressive creep curves considered here can also be efficiently represented by empirical or low-order Prony series models, especially because the data cover mainly the longer-time regime and exhibit nearly smooth trends on the logarithmic time scale. Nevertheless, the fractional Zener model and Model 13 maintained consistently high $R^{2}$ values with a limited number of parameters, showing stable descriptive capability across all three compressive creep conditions.

#### 4.2.2. Creep Compliance of Solid Propellant (NEPE)

The creep data for solid propellants were obtained from Zhang et al. [40], who investigated the creep behavior of NEPE under different loads and loading durations. They reported that the yield stress of NEPE was approximately 0.2−0.25 MPa. Accordingly, creep data under a load of 0.15 MPa with a loading duration of about $10^{6}\;s$ were selected for analysis. Due to the absence of data in the glassy region ($t\;<\;10^{0}\;s$), 3-term and 4-term Prony series were employed to fit the creep data. The upper bounds for all non-fractional-order parameters of fractional viscoelastic models were set to $10^{6}$.

**Figure 10:**
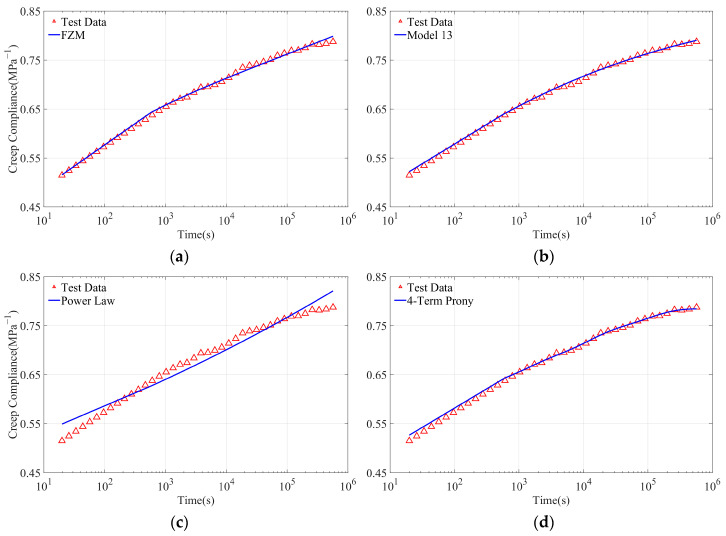
Fitting of creep data for NEPE propellant using fractional viscoelastic models and classical models: (**a**) fractional Zener model; (**b**) Model 13; (**c**) power-law model; (**d**) 4-term Prony series.

**Figure 11:**
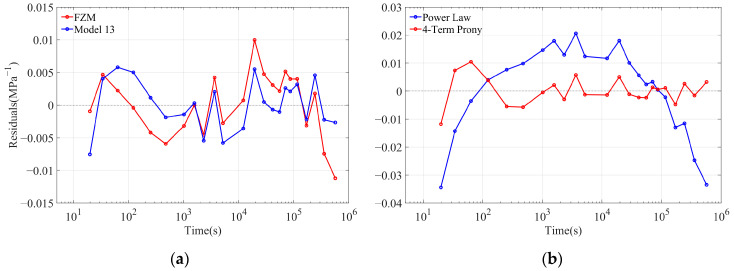
Residuals versus time of creep data for NEPE propellant using fractional viscoelastic models and classical models: (**a**) fractional Zener model and Model 13; (**b**) power-law model and 4-term Prony series.

As illustrated in Figure 10a,b,d, the two fractional models and the 4-term Prony series reasonably described the creep compliance of NEPE. Table 5 shows that these models achieved high fitting accuracy, with $R^{2}\geq 0.9964$, the residual plots in Figure 11 also demonstrate the satisfactory fit achieved by these models. Among them, Model 13 achieved the highest $R^{2}$ and the lowest AIC, indicating the best balance between fitting accuracy and parameter complexity for this dataset. The fractional Zener model and the 4-term Prony series also showed satisfactory performance, whereas the power-law model showed both a lower $R^{2}$ value and a less favorable AIC. These results suggest that Model 13 is particularly suitable for the low-rate creep response of NEPE considered here.

### 4.3. Summary and Discussion of Fitting Performance

Based on the $R^{2}$ in Appendix B, as well as the residual plots and AIC values presented in Figure 5, Figure 7 and Figure 9, and 11, Table 2, Table 3, Table 4 and Table 5, several low-parameter fractional models were able to describe both creep and relaxation responses of polymer-based energetic materials with high accuracy. In particular, the fractional Zener model and Model 13 were able to describe both creep compliance and relaxation modulus within compact rheological forms with a limited number of parameters while maintaining stable performance across different time scales. Therefore, three fractional viscoelastic models, namely the fractional Zener model, the fractional Poynting–Thomson model (Model 5 in Appendix A), and Model 13, together with the same benchmark models, were used for the assessment in the next section.

The inclusion of AIC provides a more balanced comparison among models with different parameter complexities. For the relaxation datasets, the fractional Zener model and Model 13 generally showed favorable complexity-penalized performance, indicating that compact fractional models are suitable for representing broad viscoelastic relaxation behavior. For the creep datasets, the AIC results suggest that the advantage of fractional models depends on the material response and data characteristics. In the PBX compressive creep cases, the power-law model or the 4-term Prony series achieved the lowest AIC under some loading conditions, whereas the fractional models maintained consistently high $R^{2}$ values with limited parameter numbers. Therefore, the advantage of fractional viscoelastic models should not be interpreted as universal superiority over Prony series or power-law models. Their main value lies in providing compact rheological representations for both creep and relaxation responses, with good overall accuracy and reduced parameter complexity.

The fitted responses were also checked for basic physical admissibility over the time windows of the literature data. With non-negative moduli and spring-pot coefficients and fractional orders constrained within [0, 1], the fitted relaxation moduli remained positive and non-increasing, while the fitted creep compliances remained positive and non-decreasing in magnitude. These numerical checks support the physical admissibility of the selected parameter sets within the considered data range, although they do not constitute full thermodynamic proof for arbitrary loading histories.

## 5. Assessment of Extrapolation Behavior from Early-Stage Data

In this section, early-stage data were used for calibration to assess the extrapolation behavior of selected models. Specifically, the first 10% of the time span of each dataset was used for parameter identification, and the subsequent long-time response was used to evaluate extrapolation performance. The complete curves are shown in the figures for visual comparison, and the corresponding parameters and rRMSE values are provided in Appendix B.

### 5.1. Extrapolation of Relaxation Modulus

#### 5.1.1. Extrapolation of Solid Propellant Relaxation Data (PVC/AP)

**Figure 12:**
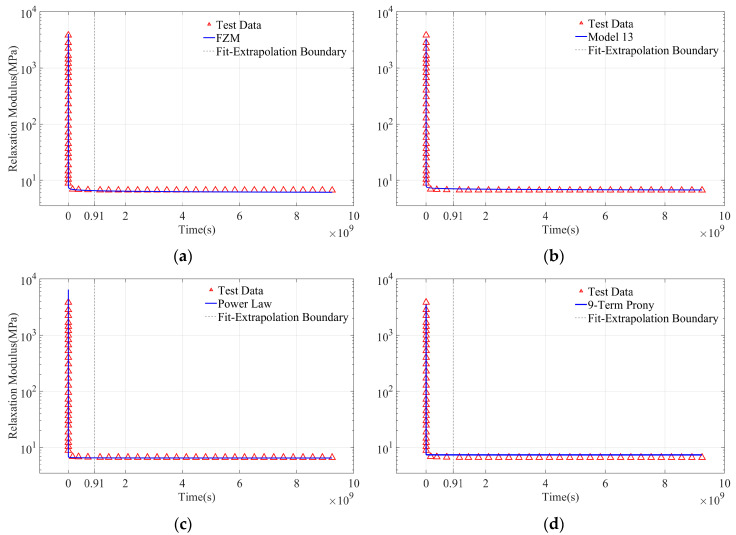
Extrapolation results of relaxation data for solid propellant (PVC/AP) using fractional viscoelastic models and classical models: (**a**) fractional Zener model; (**b**) Model 13; (**c**) power-law model; (**d**) 9-term Prony series.

In the extrapolation of the PVC/AP relaxation data, the fractional Zener model achieved $R^{2}=0.9922$ and rRMSE=5.78%, while Model 13 achieved $R^{2}=0.9915$ and rRMSE=3.45%, as shown in Figure 12 and Table 6. By contrast, although the power-law model yielded the lowest extrapolation error (rRMSE=1.63%), its relatively low coefficient of determination ($R^{2}=0.7776$) indicated that it did not adequately capture the overall shape of the relaxation curve. The 9-term Prony series achieved both a high $R^{2}$ value ($R^{2}=0.9952$) and a low rRMSE (1.88%). Overall, the two fractional viscoelastic models combined consistently high $R^{2}$ values with acceptable extrapolation errors, indicating stable extrapolation behavior within the considered dataset.

At sufficiently long times, all exponential terms in the Prony series decay to zero, causing the extrapolated curve to approach a horizontal asymptote. For polymer-based energetic materials in the rubbery state, the stress decay rate is inherently very low. The extrapolation performance of the Prony series model should be interpreted within the adopted fitting protocol, because it depends strongly on the prescribed relaxation times, the number of terms, and possible regularization strategies.

#### 5.1.2. Extrapolation of PBX Relaxation Data

**Figure 13:**
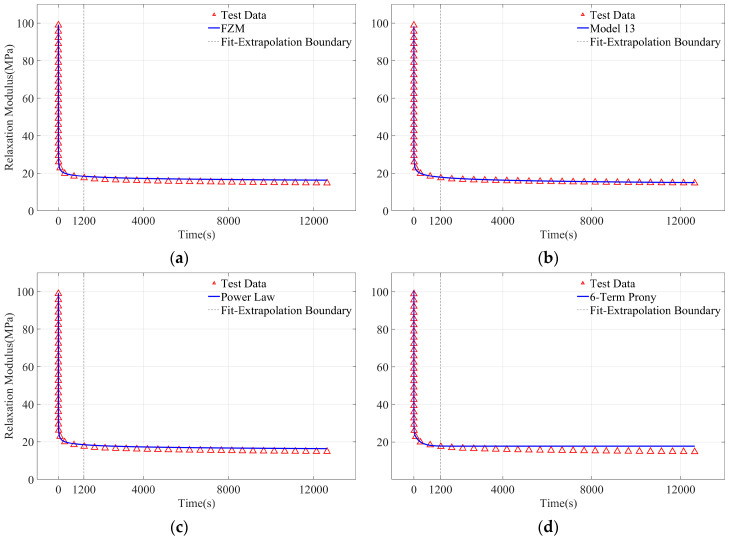
Extrapolation results of relaxation data for PBX using fractional viscoelastic models and classical models: (**a**) fractional Zener model; (**b**) Model 13; (**c**) power-law model; (**d**) 6-term Prony series.

Similarly, when the first 10% of the time span was used for parameter identification, as Table 7 indicate, both fractional models and the power-law model achieved favorable extrapolation performance for the relaxation master curve of PBX (rRMSE≤8.45%). The 6-term Prony series still achieved a high $R^{2}$ value but showed a larger quantitative extrapolation error (rRMSE=13.21%), while the lower-term Prony series exhibited unsatisfactory results (rRMSE≥33.37%). In contrast, the extrapolated curves of the two fractional models were closer to the experimental data, and the power-law model showed similar extrapolative capability, while the Prony series exhibited unstable performance. As shown in Figure 13, the extrapolated curve of the Prony series displayed a near-zero stress rate in the rubbery region, which deviated from the observed relaxation trend in the rubbery region.

### 5.2. Extrapolation of Creep Compliance

#### 5.2.1. Extrapolation of PBX Creep Data

**Figure 14:**
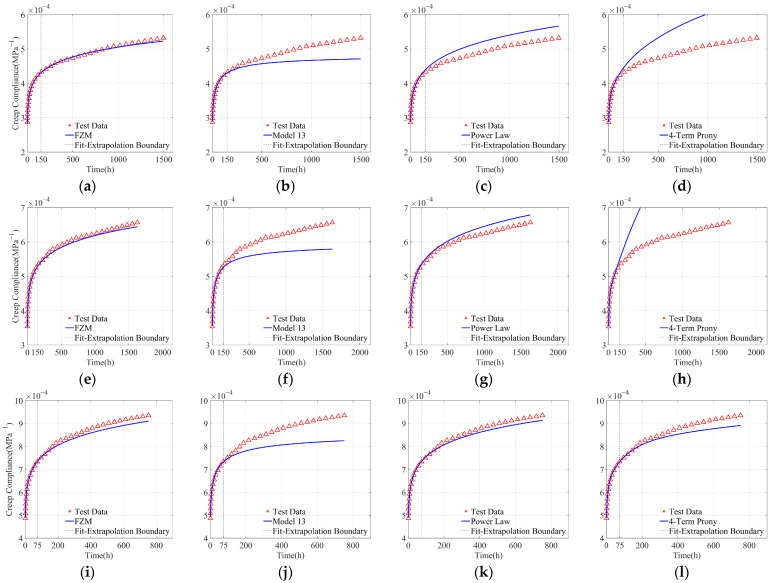
Extrapolation results for the compressive creep data of PBX under different load and temperature conditions using fractional viscoelastic and classical models: (**a**) fractional Zener model at −3 MPa and 40 °C; (**b**) Model 13 at −3 MPa and 40 °C; (**c**) power-law model at −3 MPa and 40 °C; (**d**) 4-term Prony series at −3 MPa and 40 °C; (**e**) fractional Zener model at −4 MPa and 40 °C; (**f**) Model 13 at −4 MPa and 40 °C; (**g**) power-law model at −4 MPa and 40 °C; (**h**) 4-term Prony series at −4 MPa and 40 °C; (**i**) fractional Zener model at −5 MPa and 50 °C; (**j**) Model 13 at −5 MPa and 50 °C; (**k**) power-law model at −5 MPa and 50 °C; (**l**) 4-term Prony series at −5 MPa and 50 °C.

As shown in Figure 14 and Table 8, when extrapolating the compressive creep data of PBX under loads of −3 MPa,−4 MPa, and −5 MPa, the fractional Zener model achieved the best and most stable extrapolation performance, with consistently high coefficients of determination ($R^{2}\geq 0.9943$) and low extrapolation errors (rRMSE≤1.74%) under all three conditions. The power-law model also showed reasonably good extrapolative capability, with rRMSE values ranging from 1.84% to 5.34%, although its overall stability across the three conditions remained inferior to that of the fractional Zener model. By contrast, Model 13 yielded less satisfactory results, with relatively low $R^{2}$ values ($0.8767\leq R^{2}\leq 0.9348$) and higher rRMSE values (6.73%≤rRMSE≤8.19%). Among the Prony series models, the 3-term Prony series exhibited consistently poor extrapolation performance, with negative $R^{2}$ values at −3 MPa and −4 MPa and large extrapolation errors (rRMSE≥13.08%), while the 4-term Prony series performed better but remained unstable, with rRMSE values ranging from 3.11% to 34.44%. In particular, the 4-term Prony series achieved acceptable results at −5 MPa ($R^{2}=0.9822$, rRMSE=3.11%), its extrapolation performance deteriorated markedly at the lower load levels. Notably, although Model 13 did not achieve satisfactory extrapolation performance under these three conditions, its extrapolated curves consistently exhibited relatively low steady-state creep rates.

#### 5.2.2. Extrapolation of Solid Propellant Creep Data (NEPE)

**Figure 15:**
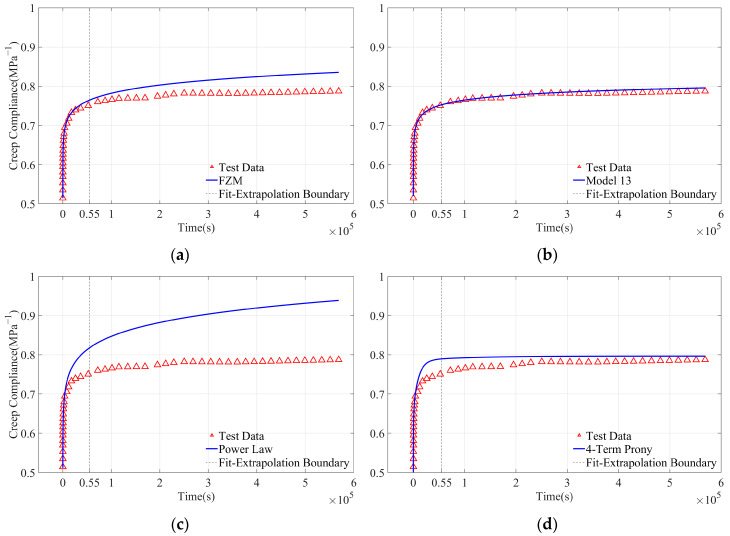
Extrapolation results for the creep data for NEPE propellant using fractional viscoelastic models and classical models: (**a**) fractional Zener model; (**b**) Model 13; (**c**) power-law model; (**d**) 4-term Prony series.

In the case of NEPE creep extrapolation, as shown in Figure 15 and Table 9, Model 13 achieved favorable extrapolation performance (rRMSE=0.56%), comparable to that of the 4-term Prony series (rRMSE=0.33%), and significantly better than the fractional Zener model (rRMSE=3.65%), while the power-law model exhibited the poorest extrapolation performance (rRMSE=13.45%). These results indicate that Model 13 is particularly suitable for materials characterized by low strain rates during the steady-state creep stage. Although the 4-term Prony series also achieved a low extrapolation error, its extrapolated curve exhibited a distinct linear trend in the steady-state creep regime, suggesting that, under the present fixed time-constant setting, the generalized Kelvin model may produce an overly linear extrapolation trend in the steady-state creep regime. By contrast, Model 13 provided both high extrapolative accuracy and a smoother description of the low-rate steady-state creep response.

### 5.3. Summary and Discussion of Extrapolation Behavior

According to Figure 12, Figure 13, Figure 14 and Figure 15, and the $R^{2}$, rRMSE in Table 6, Table 7, Table 8 and Table 9 and Appendix B, the extrapolation results indicate that the fractional Zener model and Model 13 provide different advantages depending on the material response. For relaxation data, both models gave stable extrapolation within the considered datasets. For creep data, the fractional Zener model was more suitable for cases with relatively high steady-state creep rates, whereas Model 13 performed better for low-rate creep responses. The Prony series models also showed excellent performance in some cases, especially when sufficient terms were used. However, their extrapolation behavior depended on the prescribed time constants and the number of terms. The power-law model remains useful as an engineering approximation for datasets exhibiting approximately power-law trends, but it lacks a direct rheological analog for unified creep-relaxation interpretation.

Since only the first 10% of the time span was used for calibration, the extrapolation assessment corresponds to an approximately one-order-of-magnitude extension in the time window within the available experimental curves. This setting provides a practical test of early-stage data extrapolation, although it should not be interpreted as independent experimental validation.

It should also be noted that the present assessments were restricted to linear viscoelastic models. The relaxation datasets considered in this study were master curves constructed using the time–temperature superposition principle, which is commonly applied within the small-strain linear viscoelastic framework. However, because the published datasets do not always provide multi-level creep or relaxation curves, the linear viscoelastic assumption cannot be independently verified for all cases, especially under relatively high compressive loading conditions. Possible nonlinear viscoelastic effects at higher stress or strain levels may lead to load-dependent parameters and reduce the reliability of extrapolation outside the calibrated loading condition.

The fractional parameters identified in this study should be interpreted as phenomenological quasi-properties rather than direct microstructural descriptors. The fractional orders may reflect the combined effects of binder viscoelasticity, particle–binder interfacial interactions, particle-packing heterogeneity, and distributed relaxation mechanisms. Nevertheless, the present macroscopic creep and relaxation data do not allow a definitive correspondence between individual fractional parameters and specific microstructural features to be established. Establishing such a relationship would require additional microstructural characterization and multiscale mechanical analysis.

In addition, an in-house PBX creep dataset was further analyzed, and the results are provided in Appendix C. The supplementary results show similar trends in model performance and provide an additional consistency check for the applicability of the selected fractional models to PBX creep data. However, this supplementary analysis should not be interpreted as complete independent validation over all material systems, loading conditions, or experimental uncertainties.

## 6. Conclusions

This study compared low-parameter fractional viscoelastic models for describing and extrapolating creep and stress relaxation behaviors of polymer-based energetic materials. Fourteen candidate models composed of springs and spring-pot elements were considered under controlled parameter complexity, and their performance was evaluated using PBX and solid propellant datasets. The main conclusions are as follows:

(1) Among the 14 candidate models, the fractional Zener model and Model 13 showed the most favorable overall performance for the datasets considered. These two models can describe both creep compliance and stress relaxation modulus within compact six-parameter rheological forms, with coefficients of determination generally exceeding 0.99. Compared with high-order Prony series models in the relaxation cases, they achieved comparable fitting accuracy with lower parameter complexity.
(2) The combined Talbot–Gray Wolf parameter identification procedure provided a practical parameter identification route for fractional viscoelastic models without analytical time-domain solutions. With the numerical settings specified in this study, the procedure successfully identified parameters for the candidate models. Repeated optimization results in Appendix D further indicate that the identified parameters remained within bounded ranges for the representative case considered.
(3) When the first 10% of the time span was used for calibration, the selected fractional models showed reasonable extrapolation behavior over an approximately one-order-of-magnitude longer time window within the available experimental curves. The rRMSE values of the fractional Zener model and Model 13 were below 8.5% in the reported extrapolation cases, and below 2% under their respective suitable conditions.
(4) The comparison with Prony series and power-law models shows that compact fractional models provide useful alternatives for representing broad viscoelastic response behavior. Prony series models remain standard tools in linear viscoelasticity, but their extrapolation behavior depends on the prescribed relaxation or retardation times and the number of terms. The power-law model can still serve as an engineering approximation for approximately power-law responses, although it lacks a direct rheological analog for unified creep-relaxation interpretation.
(5) In addition to published datasets, an in-house PBX creep dataset was analyzed as a supplementary check in Appendix C. The supplementary results showed similar trends in model performance and provided an additional consistency check for the applicability of the selected fractional models to PBX creep analysis.

The present work should be interpreted as a comparative model assessment based mainly on published datasets, rather than as complete independent experimental validation. Future work should therefore combine controlled multi-level experiments, normalized relaxation or creep curve comparisons, nonlinear viscoelastic modeling, and broader parameter identifiability analyses to further assess the model transferability and applicability.

## Figures and Tables

**Table 1:** Numerical settings for parameter identification.

Item	Setting
Numerical inverse Laplace method	Talbot method
Number of Talbot truncation terms	64
Optimizer	Gray Wolf Optimizer
Population size	40
Maximum iterations	500
Random seed	2025
Lower bounds of all parameters	0
Fractional-order bounds	[0, 1]
Termination criterion	Maximum iteration number
Objective function	Sum of squared relative errors

**Table 2:** Coefficients of determination and Akaike information criterion corresponding to the compared models for the solid propellant (PVC/AP) relaxation data.

Model	FZM	Model 13	Fang et al.’s Fractional Model [22]	Power Law	9-Term Prony
$R^{2}$	0.9988	0.9919	0.9960	0.7772	0.9976
AIC	340.7258	429.1384	398.4827	575.3599	399.7494

**Table 3:** Coefficients of determination and Akaike information criterion corresponding to the compared models for the PBX relaxation data.

Model	FZM	Model 13	Power Law	4-Term Prony	5-Term Prony	6-Term Prony
$R^{2}$	0.9997	0.9999	0.9990	0.9813	0.9908	0.9977
AIC	−58.6022	−100.9052	−8.3533	140.1445	110.9688	48.6924

**Table 4:** Coefficients of determination and Akaike information criterion corresponding to the compared models for the PBX compression creep data.

Model	FZM	Model 13	Power Law	3-Term Prony	4-Term Prony
$R^{2}(-3\;\mathrm{MPa})$	0.9982	0.9987	0.9984	0.9978	0.9983
AIC(−3 MPa)	−444.0164	−449.7906	−452.8295	−444.7925	−446.9092
$R^{2}(-4\;\mathrm{MPa})$	0.9994	0.9991	0.9995	0.9988	0.9995
AIC(−4 MPa)	−636.3960	−625.0108	−644.0084	−621.7003	−639.5528
$R^{2}(-5\;\mathrm{MPa})$	0.9994	0.9992	0.9992	0.9971	0.9996
AIC(−5 MPa)	−390.1263	−385.3738	−392.8688	−369.2515	−400.2626

**Table 5:** Coefficients of determination and Akaike information criterion corresponding to the compared models for the NEPE propellant creep data.

Model	FZM	Model 13	Power Law	3-Term Prony	4-Term Prony
$R^{2}$	0.9964	0.9979	0.9626	0.9841	0.9965
AIC	−233.8027	−245.9398	−185.7660	−203.4257	−236.2729

**Table 6:** Extrapolation performance of the compared models for the solid propellant (PVC/AP) relaxation data.

Model	FZM	Model 13	Power Law	9-Term Prony
$R^{2}$	0.9922	0.9915	0.7776	0.9952
rRMSE	5.78%	3.45%	1.63%	1.88%

**Table 7:** Extrapolation performance of the compared models for the PBX relaxation data.

Model	FZM	Model 13	Power Law	4-Term Prony	5-Term Prony	6-Term Prony
$R^{2}$	0.9993	0.9998	0.9993	−2.0537	0.9852	0.9969
rRMSE	8.43%	2.37%	8.45%	100.11%	33.37%	13.21%

**Table 8:** Extrapolation performance of the compared models for the PBX compression creep data.

Model	FZM	Model 13	Power Law	3-Term Prony	4-Term Prony
$R^{2}(-3\;\mathrm{MPa})$	0.9979	0.9348	0.9587	−0.5730	0.6832
rRMSE(−3 MPa)	0.95%	6.73%	5.34%	33.13%	14.85%
$R^{2}(-4\;\mathrm{MPa})$	0.9969	0.9099	0.9894	−0.4400	−1.0808
rRMSE(−4 MPa)	1.27%	7.16%	2.45%	28.65%	34.44%
$R^{2}(-5\;\mathrm{MPa})$	0.9943	0.8767	0.9937	0.6837	0.9822
rRMSE(−5 MPa)	1.74%	8.19%	1.84%	13.08%	3.11%

**Table 9:** Extrapolation performance of the compared models for the NEPE propellant creep data.

Model	FZM	Model 13	Power Law	3-Term Prony	4-Term Prony
$R^{2}$	0.9563	0.9979	0.3488	0.8690	0.9965
rRMSE	3.65%	0.56%	13.45%	4.19%	0.33%

**Figure A1:**
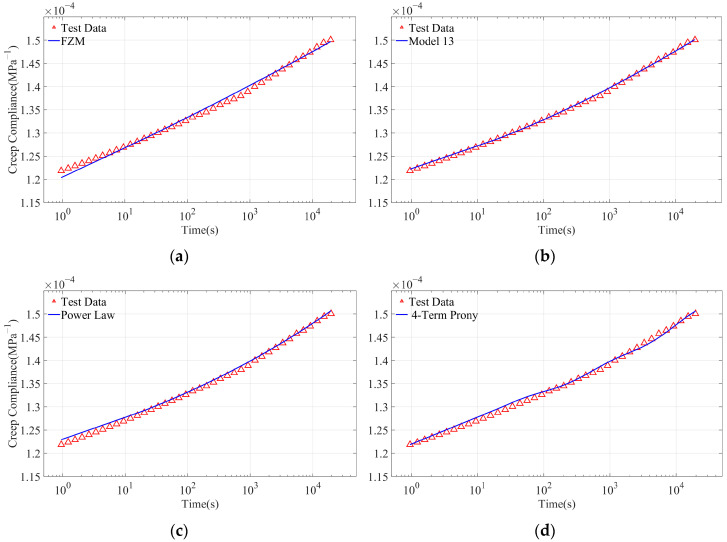
Fitting of creep data for PBX using fractional viscoelastic models and classical models (test data): (**a**) fractional Zener model; (**b**) Model 13; (**c**) power-law model; (**d**) 4-term Prony series.

**Figure A2:**
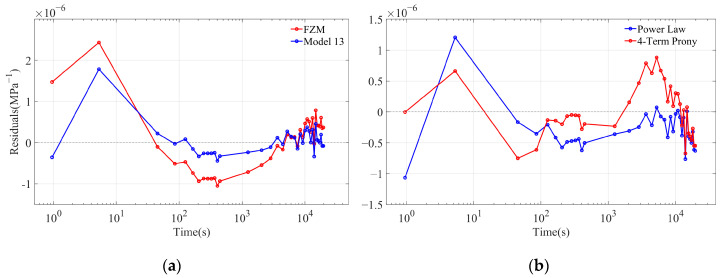
Residuals versus time of creep data for PBX using fractional viscoelastic models and classical models (test data): (**a**) fractional Zener model and Model 13; (**b**) power-law model and 4-term Prony series.

**Figure A3:**
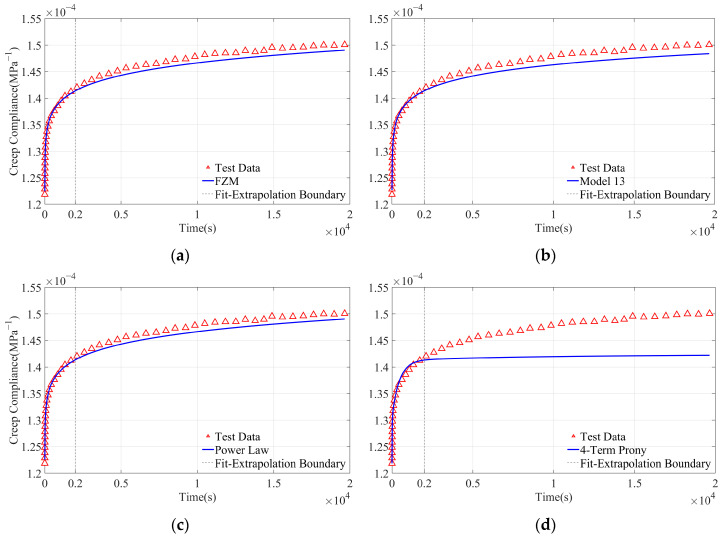
Extrapolation results for the creep data for PBX using fractional viscoelastic models and classical models (test data): (**a**) fractional Zener model; (**b**) Model 13; (**c**) power-law model; (**d**) 4-term Prony series.

**Figure A4:**
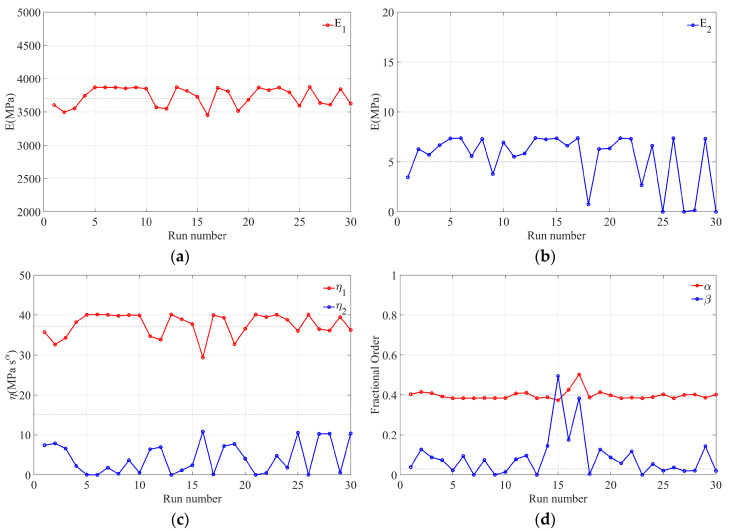
Distributions of the identified parameters obtained from 30 independent optimization runs for fitting Model 13 to the PVC/AP relaxation master curve (**a**) $E_{1}$; (**b**) $E_{2}$; (**c**) $\eta_{1}$ and $\eta_{2}$; (**d**) α and β.

**Figure A5:**
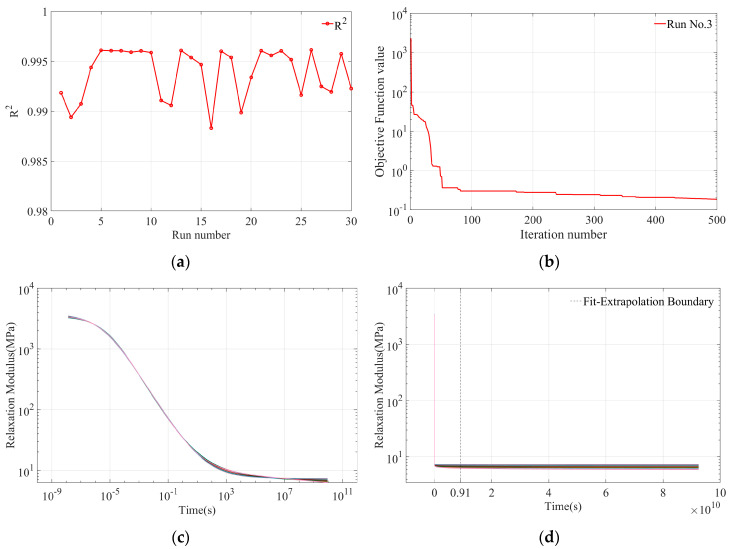
Response-level stability and convergence behavior obtained from 30 independent optimization runs for fitting Model 13 to the PVC/AP relaxation master curve: (**a**) $R^{2}$ values; (**b**) convergence history of the best objective value in the third independent run; (**c**) fitted curves obtained from the 30 independent runs; (**d**) extrapolated curves over the approximately one-order-of-magnitude longer time window.

## Data Availability

The data used in this study are available from the cited references and the supplementary tables in the Appendix.

## Appendix A. Candidate Fractional Viscoelastic Models

This appendix summarizes the 14 candidate fractional viscoelastic models considered in this study. For each model, the mechanical analog, differential constitutive equation, creep compliance, and relaxation modulus are provided, as shown in Table A1. These models were constructed under the complexity constraint adopted in the main text, and degenerate configurations reducible to simpler equivalent forms were excluded.

**Table A1:** Summary of the 14 fractional viscoelastic models: mechanical analogs, differential constitutive relations, creep compliances, and relaxation moduli.

Model	Differential Constitutive Equation	Creep Compliance	Relaxation Modulus
**	$\sigma +\frac{\eta_{1}}{\eta_{2}}D^{\alpha -\beta }\sigma =\eta_{1}D^{\alpha }\varepsilon$	$J\left(t\right)=\frac{1}{\eta_{1}}\frac{t^{\alpha }}{\Gamma (1+\alpha )}+\frac{1}{\eta_{2}}\frac{t^{\beta }}{\Gamma (1+\beta )}$	$G\left(t\right)=\eta_{2}\cdot t^{-\beta }\cdot E_{\alpha -\beta ,1-\beta }(-\frac{\eta_{2}}{\eta_{1}}\cdot t^{\alpha -\beta })$
Model 1			
	$\sigma =\eta_{1}D^{\alpha }\varepsilon +\eta_{2}D^{\beta }\varepsilon$	$J\left(t\right)=\frac{1}{\eta_{1}}\cdot t^{\alpha }\cdot E_{\alpha -\beta ,1+\alpha }(-\frac{\eta_{2}}{\eta_{1}}\cdot t^{\alpha -\beta })$	$G\left(t\right)=\eta_{1}\cdot \frac{t^{-\alpha }}{\Gamma \left(1-\alpha \right)}+\eta_{2}\cdot \frac{t^{-\beta }}{\Gamma \left(1-\beta \right)}$
Model 2			
	$\sigma +\frac{\eta_{1}}{\eta_{2}}D^{\alpha -\beta }\sigma +\frac{\eta_{1}}{\eta_{3}}D^{\alpha -\gamma }\sigma =\eta_{1}D^{\alpha }\varepsilon$	$J\left(t\right)=\frac{1}{\eta_{1}}\frac{t^{\alpha }}{\Gamma (1+\alpha )}+\frac{1}{\eta_{2}}\frac{t^{\beta }}{\Gamma (1+\beta )}+\frac{1}{\eta_{3}}\frac{t^{\gamma }}{\Gamma (1+\gamma )}$	$G\left(s\right)=\frac{\eta_{1}\eta_{2}\eta_{3}\cdot s^{\alpha +\beta +\gamma }}{\eta_{1}\eta_{2}\cdot s^{\alpha +\beta +1}+\eta_{1}\eta_{3}\cdot s^{\alpha +\gamma +1}+\eta_{2}\eta_{3}\cdot s^{\beta +\gamma +1}}$
Model 3			
	$\sigma =\eta_{1}D^{\alpha }\varepsilon +\eta_{2}D^{\beta }\varepsilon +\eta_{3}D^{\gamma }\varepsilon$	$J\left(s\right)=\frac{1}{\eta_{1}\cdot s^{\alpha +1}+\eta_{2}\cdot s^{\beta +1}+\eta_{3}\cdot s^{\gamma +1}}$	$G\left(s\right)=\eta_{1}\cdot \frac{t^{-\alpha }}{\Gamma \left(1-\alpha \right)}+\eta_{2}\cdot \frac{t^{-\beta }}{\Gamma \left(1-\beta \right)}+\eta_{3}\cdot \frac{t^{-\gamma }}{\Gamma \left(1-\gamma \right)}$
Model 4			
	$\sigma +\frac{\eta_{2}}{\eta_{1}}D^{\beta -\alpha }\sigma +\frac{\eta_{3}}{\eta_{1}}D^{\gamma -\alpha }\sigma =\eta_{2}D^{\beta }\varepsilon +\eta_{3}D^{\gamma }\varepsilon$	$J\left(t\right)=\frac{1}{\eta_{1}}\frac{t^{\alpha }}{\Gamma (1+\alpha )}+\frac{1}{\eta_{2}}\cdot t^{\beta }\cdot E_{\beta -\gamma ,1+\beta }(-\frac{\eta_{3}}{\eta_{2}}\cdot t^{\beta -\gamma })$	$G\left(s\right)=\frac{\eta_{1}\eta_{2}\cdot s^{\alpha +\beta }+\eta_{1}\eta_{3}\cdot s^{\alpha +\gamma }}{\eta_{1}\cdot s^{\alpha +1}+\eta_{2}\cdot s^{\beta +1}+\eta_{3}\cdot s^{\gamma +1}}$
Model 5			
	$\sigma +\frac{\eta_{2}}{\eta_{1}}D^{\beta -\alpha }\sigma =\eta_{2}D^{\beta }\varepsilon +\eta_{3}D^{\gamma }\varepsilon +\frac{\eta_{2}\eta_{3}}{\eta_{1}}D^{\beta +\gamma -\alpha }\varepsilon$	$J\left(s\right)=\frac{\eta_{1}\cdot s^{\alpha }+\eta_{2}\cdot s^{\beta }}{\eta_{1}\eta_{2}\cdot s^{\alpha +\beta +1}+\eta_{1}\eta_{3}\cdot s^{\alpha +\gamma +1}+\eta_{2}\eta_{3}\cdot s^{\beta +\gamma +1}}$	$G\left(t\right)=\eta_{2}\cdot t^{-\beta }\cdot E_{\alpha -\beta ,1-\beta }\left(-\frac{\eta_{2}}{\eta_{1}}\cdot t^{\alpha -\beta }\right)+\eta_{3}\cdot \frac{t^{-\gamma }}{\Gamma \left(1-\gamma \right)}$
Model 6			
	$\sigma +\frac{E_{1}\eta_{1}+E_{2}\eta_{1}}{E_{1}\eta_{1}+E_{2}\eta_{2}}D^{\alpha -\beta }\sigma +\frac{\eta_{1}\eta_{2}}{E_{1}\eta_{1}+E_{2}\eta_{2}}D^{\alpha }\sigma \;=\frac{E_{1}E_{2}\eta_{2}}{E_{1}\eta_{1}+E_{2}\eta_{2}}\varepsilon +\frac{E_{1}\eta_{1}\eta_{2}}{E_{1}\eta_{1}+E_{2}\eta_{2}}D^{\alpha }\varepsilon +\frac{E_{1}E_{2}\eta_{1}}{E_{1}\eta_{1}+E_{2}\eta_{2}}D^{\alpha -\beta }\varepsilon$	$J\left(s\right)=\frac{{(E}_{1}\eta_{1}+E_{2}\eta_{1})\cdot s^{\alpha }+\left(E_{1}\eta_{1}+E_{2}\eta_{2}\right)\cdot s^{\beta }+\eta_{1}\eta_{2}\cdot s^{\alpha +\beta }}{E_{1}E_{2}\eta_{1}\cdot s^{\alpha +1}+E_{1}E_{2}\eta_{2}\cdot s^{\beta +1}+E_{1}\eta_{1}\eta_{2}\cdot s^{\alpha +\beta +1}}$	$G\left(s\right)=\frac{E_{1}E_{2}\eta_{1}\cdot s^{\alpha }+E_{1}E_{2}\eta_{2}\cdot s^{\beta }+E_{1}\eta_{1}\eta_{2}\cdot s^{\alpha +\beta }}{{(E}_{1}\eta_{1}+E_{2}\eta_{1})\cdot s^{\alpha +1}+\left(E_{1}\eta_{1}+E_{2}\eta_{2}\right)\cdot s^{\beta +1}+\eta_{1}\eta_{2}\cdot s^{\alpha +\beta +1}}$
Model 7			
	$\sigma +\frac{\eta_{1}}{E_{2}}D^{\alpha }\sigma +\left(\frac{\eta_{2}}{E_{1}}+\frac{\eta_{2}}{E_{2}}\right)D^{\beta }\sigma +\frac{\eta_{1}\eta_{2}}{E_{1}E_{2}}D^{\alpha +\beta }\sigma =\eta_{2}D^{\beta }\varepsilon +\frac{\eta_{1}\eta_{2}}{E_{1}E_{2}}D^{\alpha +\beta }\varepsilon$	$J\left(s\right)=\frac{E_{1}E_{2}+E_{1}\eta_{1}\cdot s^{\alpha }+\left(E_{1}\eta_{2}+E_{2}\eta_{2}\right)\cdot s^{\beta }+\eta_{1}\eta_{2}\cdot s^{\alpha +\beta }}{E_{1}E_{2}\eta_{2}\cdot s^{\beta +1}+E_{1}\eta_{1}\eta_{2}\cdot s^{\alpha +\beta +1}}$	$G\left(s\right)=\frac{E_{1}E_{2}\eta_{2}\cdot s^{\beta }+E_{1}\eta_{1}\eta_{2}\cdot s^{\alpha +\beta }}{E_{1}E_{2}\cdot s+E_{1}\eta_{1}\cdot s^{\alpha +1}+\left(E_{1}\eta_{2}+E_{2}\eta_{2}\right)\cdot s^{\beta +1}+\eta_{1}\eta_{2}\cdot s^{\alpha +\beta +1}}$
Model 8			
	$\sigma +\frac{\eta_{2}}{E_{2}}D^{\beta }\sigma =E_{1}\varepsilon +\eta_{1}D^{\alpha }\varepsilon +\left(\frac{E_{1}}{E_{2}}\eta_{2}+\eta_{2}\right)D^{\beta }\varepsilon +\frac{\eta_{1}\eta_{2}}{E_{2}}D^{\alpha +\beta }\varepsilon$	$J\left(s\right)=\frac{E_{2}+\eta_{2}\cdot s^{\beta }}{E_{1}E_{2}\cdot s+E_{2}\eta_{1}\cdot s^{\alpha +1}+\left(E_{1}\eta_{2}+E_{2}\eta_{2}\right)\cdot s^{\beta +1}+\eta_{1}\eta_{2}\cdot s^{\alpha +\beta +1}}$	$G\left(t\right)=E_{1}+\eta_{1}\frac{t^{-\alpha }}{\Gamma (1-\alpha )}+E_{2}\cdot E_{\beta ,1}(-\frac{E_{2}}{\eta_{2}}t^{\beta })$
Model 9			
	$\sigma +\frac{\eta_{1}}{E_{1}}D^{\alpha }\sigma +\left(\frac{\eta_{2}}{E_{2}}+\frac{1}{E_{1}}\right)D^{\beta }\sigma +\frac{\eta_{1}\eta_{2}}{E_{1}E_{2}}D^{\alpha +\beta }\sigma =\eta_{1}D^{\alpha }\varepsilon +D^{\beta }\varepsilon +\frac{\eta_{1}\eta_{2}}{E_{2}}D^{\alpha +\beta }\varepsilon$	$J\left(s\right)=\frac{E_{1}E_{2}+E_{2}\eta_{1}\cdot s^{\alpha }+\left(E_{1}\eta_{2}+E_{2}\right)\cdot s^{\beta }+\eta_{1}\eta_{2}\cdot s^{\alpha +\beta }}{E_{1}E_{2}\eta_{1}\cdot s^{\alpha +1}+E_{1}E_{2}\cdot s^{\beta +1}+E_{1}\eta_{1}\eta_{2}\cdot s^{\alpha +\beta +1}}$	$G\left(s\right)=\frac{E_{1}E_{2}\eta_{1}\cdot s^{\alpha }+E_{1}E_{2}\cdot s^{\beta }+E_{1}\eta_{1}\eta_{2}\cdot s^{\alpha +\beta }}{E_{1}E_{2}\cdot s+E_{2}\eta_{1}\cdot s^{\alpha +1}+\left(E_{1}\eta_{2}+E_{2}\right)\cdot s^{\beta +1}+\eta_{1}\eta_{2}\cdot s^{\alpha +\beta +1}}$
Model 10			
	$\sigma +\frac{\eta_{1}}{E_{1}}D^{\alpha }\sigma +\frac{\eta_{2}}{E_{2}}D^{\beta }\sigma +\frac{\eta_{1}\eta_{2}}{E_{1}E_{2}}D^{\alpha +\beta }\sigma \;=\eta_{1}D^{\alpha }\varepsilon +\eta_{2}D^{\beta }\varepsilon +(\frac{\eta_{1}\eta_{2}}{E_{1}}+\frac{\eta_{1}\eta_{2}}{E_{2}})D^{\alpha +\beta }\varepsilon$	$J\left(s\right)=\frac{E_{1}E_{2}+E_{2}\eta_{1}\cdot s^{\alpha }+E_{1}\eta_{2}\cdot s^{\beta }+\eta_{1}\eta_{2}\cdot s^{\alpha +\beta }}{E_{1}E_{2}\eta_{1}\cdot s^{\alpha +1}+E_{1}E_{2}\eta_{2}\cdot s^{\beta +1}+(E_{1}\eta_{1}\eta_{2}+E_{2}\eta_{1}\eta_{2})\cdot s^{\alpha +\beta +1}}$	$G\left(t\right)=E_{1}\cdot E_{\alpha ,1}\left(-\frac{E_{1}}{\eta_{1}}t^{\alpha }\right)+E_{2}\cdot E_{\beta ,1}\left(-\frac{E_{2}}{\eta_{2}}t^{\beta }\right)$
Model 11			
	$\sigma +\frac{\eta_{1}}{E_{1}{+E}_{2}}D^{\alpha }\sigma +\frac{\eta_{2}}{E_{1}{+E}_{2}}D^{\beta }\sigma \;=\frac{E_{1}E_{2}}{E_{1}{+E}_{2}}\varepsilon +\frac{E_{2}\eta_{1}}{E_{1}{+E}_{2}}D^{\alpha }\varepsilon +\frac{E_{1}\eta_{2}}{E_{1}{+E}_{2}}D^{\beta }\varepsilon +\frac{\eta_{1}\eta_{2}}{E_{1}{+E}_{2}}D^{\alpha +\beta }\varepsilon$	$J\left(t\right)=\frac{1}{\eta_{1}}\cdot t^{\alpha }\cdot E_{\alpha ,1+\alpha }\left(-\frac{E_{1}}{\eta_{1}}t^{\alpha }\right)+\frac{1}{\eta_{2}}\cdot t^{\beta }\cdot E_{\beta ,1+\beta }\left(-\frac{E_{2}}{\eta_{2}}t^{\beta }\right)$	$G\left(s\right)=\frac{E_{1}E_{2}+E_{2}\eta_{1}\cdot s^{\alpha }+E_{1}\eta_{2}\cdot s^{\beta }+\eta_{1}\eta_{2}\cdot s^{\alpha +\beta }}{\left(E_{1}{+E}_{2}\right)\cdot s+\eta_{1}\cdot s^{\alpha +1}+\eta_{2}\cdot s^{\beta +1}}$
Model 12			
	$\sigma +\frac{\eta_{1}}{E_{1}{+E}_{2}}D^{\alpha }\sigma +\frac{\eta_{2}}{E_{1}{+E}_{2}}D^{\beta }\sigma =\frac{E_{1}E_{2}}{E_{1}{+E}_{2}}\varepsilon +\frac{E_{1}\eta_{1}}{E_{1}{+E}_{2}}D^{\alpha }\varepsilon +\frac{E_{1}\eta_{2}}{E_{1}{+E}_{2}}D^{\beta }\varepsilon$	$J\left(s\right)=\frac{E_{1}{+E}_{2}+\eta_{1}\cdot s^{\alpha }+\eta_{2}\cdot s^{\beta }}{E_{1}E_{2}\cdot s+E_{1}\eta_{1}\cdot s^{\alpha +1}+E_{1}\eta_{2}\cdot s^{\beta +1}}$	$G\left(s\right)=\frac{E_{1}E_{2}+E_{1}\eta_{1}\cdot s^{\alpha }+E_{1}\eta_{2}\cdot s^{\beta }}{{(E}_{1}{+E}_{2})\cdot s+\eta_{1}\cdot s^{\alpha +1}+\eta_{2}\cdot s^{\beta +1}}$
Model 13			
	$\sigma +\left(\frac{\eta_{1}}{E_{1}}+\frac{\eta_{1}}{E_{2}}\right)D^{\alpha }\sigma +\frac{\eta_{2}}{E_{2}}D^{\beta }\sigma +\frac{\eta_{1}\eta_{2}}{E_{1}E_{2}}D^{\alpha +\beta }\sigma =\eta_{2}D^{\beta }\varepsilon +(\frac{\eta_{1}\eta_{2}}{E_{1}}+\frac{\eta_{1}\eta_{2}}{E_{2}})D^{\alpha +\beta }\varepsilon$	$J\left(t\right)=\frac{1}{E_{2}}-\frac{E_{1}}{E_{2}\left(E_{1}{+E}_{2}\right)}\cdot E_{\alpha ,1}\left(-\frac{E_{1}E_{2}}{\eta_{1}\left(E_{1}{+E}_{2}\right)}\cdot t^{\alpha }\right)+\frac{1}{\eta_{2}}\frac{t^{\beta }}{\Gamma (1+\beta )}$	$G\left(s\right)=\frac{E_{1}E_{2}\eta_{2}\cdot s^{\beta }+(E_{1}\eta_{1}\eta_{2}+E_{2}\eta_{1}\eta_{2})\cdot s^{\alpha +\beta }}{E_{1}E_{2}\cdot s+\left(E_{1}\eta_{1}+E_{2}\eta_{1}\right)\cdot s^{\alpha +1}+E_{1}\eta_{2}\cdot s^{\beta +1}+\eta_{1}\eta_{2}\cdot s^{\alpha +\beta +1}}$
Model 14			

## Appendix B. Fitted Parameters and Extrapolation Metrics

This appendix provides the identified parameters and error metrics for the fitting and extrapolation analyses reported in Section 4 and Section 5. Table A2, Table A3, Table A4, Table A5, Table A6 and Table A7 summarize the fitting parameters and fitting-related indicators, including $R^{2}$ and AIC. Table A8, Table A9, Table A10, Table A11, Table A12 and Table A13 provide the parameters and error metrics for early-stage-data extrapolation, including $R^{2}$ and rRMSE. The AIC values should be compared only among models fitted to the same dataset. The parameters of the Prony series are wrapped over multiple lines within the same table row for compact presentation.

**Table A2:** Optimal fitting parameters of the 14 fractional viscoelastic models, the power-law model, and the 9-term Prony series for the relaxation master curve of PVC/AP solid propellant [37].

Model	AIC	$R^{2}$	Para 1	Para 2	Para 3	Para 4	Para 5	Para 6
Model 1	541.8171	0.8971	61.5072	881.9475	0.2605	0.2604		
Model 2	577.5084	0.7765	6.5723	43.2012	0.0007	0.2902		
Model 3	546.0231	0.8967	924.9941	624.0927	67.9075	0.2604	0.2602	0.2608
Model 4	581.4085	0.7770	43.0216	0.0663	6.6525	0.2904	0.0015	0.0021
Model 5	284.0778	0.9997	948.1050	35.0225	12.1768	0.0835	0.4440	0.0282
Model 6	340.7258	0.9988	34.6308	748.9970	11.8259	0.4520	0.0984	0.0281
Model 7	411.3984	0.9945	3793.0678	29.5127	248.7318	56.0490	0.5861	0.3645
Model 8	321.9587	0.9992	5157.6970	8.7728	40.0374	911.3084	0.4151	0.1648
Model 9	378.3898	0.9973	6.9960	1797.1166	21.830	17.4119	0.2669	0.4823
Model 10	560.1167	0.8596	2140.6713	84.0406	36.6255	0	0.1155	0.8383
Model 11	416.5537	0.9938	3699.8657	9.0001	37.8533	291.0282	0.3942	0.1152
Model 12	336.0537	0.9989	7.4323	2832.7098	39.9594	178.9326	0.3909	0.1105
Model 13	429.1384	0.9919	3604.6836	3.4557	35.7143	7.4088	0.4034	0.0394
Model 14	381.2324	0.9971	8059.9075	7.3914	40.0255	5744.9143	0.3862	0.0162
Power-law	575.3599	0.7772	6.5061	33.7519	0.290			
9-term Prony	399.7494	0.9976	6.5223	1029.4410	1022.0450	919.8531	433.7441	120.7088
			30.9259	7.6280	2.2850	0.7552	$2.82\times 10^{-7}$	$3.73\times 10^{-6}$
			$8.43\times 10^{-5}$	$2.26\times 10^{-3}$	$5.01\times 10^{-2}$	2.0331	337.0353	$1.02\times 10^{5}$
			$9.24\times 10^{8}$					

**Table A3:** Optimal fitting parameters of the 14 fractional viscoelastic models, the power-law model, and the Prony series for the relaxation master curve of PBX [38].

Model	AIC	$R^{2}$	Para 1	Para 2	Para 3	Para 4	Para 5	Para 6
Model 1	130.3821	0.9812	632.4018	42.1061	0.1085	0.1080		
Model 2	3.1208	0.9987	33.7587	2.9727	0.0849	0.3564		
Model 3	135.4074	0.9808	2734.2861	3000	40.6123	0.0815	0.0960	0.1084
Model 4	−16.7562	0.9992	26.0552	7.4669	3.0830	0.1068	0.0267	0.3342
Model 5	−93.4971	0.9999	51.7387	22.6488	96.8166	0.0994	0.4861	0.0456
Model 6	−58.6022	0.9997	51.5098	6.4289	30.6852	0.1145	0.3348	0.0742
Model 7	−2.5427	0.9990	816.0819	30.4151	4082.9142	46.0053	0.2113	0.1671
Model 8	−79.7875	0.9998	523.1211	40.5835	25.6977	93.9598	0.2675	0.1402
Model 9	−8.3922	0.9991	8.6242	820.5230	28.1076	0.0797	0.1464	0.7192
Model 10	135.1844	0.9809	2996.5112	1013.9331	39.1510	217.3957	0.1115	0.0204
Model 11	137.7317	0.9798	3000	0.0426	40.0311	31.8444	0.1094	0.2183
Model 12	−2.0418	0.9990	9.5311	2535.5229	27.6147	612.0193	0.1562	0.6424
Model 13	−100.9052	0.9999	161.396	0.0193	39.9652	6.7863	0.0915	0.4289
Model 14	−73.1660	0.9998	360.649	18.6611	19.9801	915.0963	0.2227	0.2978
Power-law	−8.3533	0.9990	9.8466	23.9768	0.1594			
4-term Prony	140.1445	0.9813	14.9470	51.5123	30.6295	8.7030	12.0886	0.0010
			0.1	10	1000			
5-term Prony	110.9688	0.9908	12.4634	210.2223	38.6487	16.6121	9.0595	6.1657
			${1.26\times 10}^{-4}$	${1.19\times 10}^{-2}$	1.1220	${1.06\times 10}^{2}$	10,000	
6-term Prony	48.6924	0.9977	13.2688	134.096	36.0004	19.9881	8.7767	7.1742
			4.4916	${1.26\times 10}^{-4}$	${4.79\times 10}^{-3}$	0.1820	6.9183	$2.63\times 10^{2}$
			10,000					

**Table A4:** Optimal fitting parameters of the 14 fractional viscoelastic models, the power-law model, and the Prony series for the compressive creep data of PBX at −3 MPa and 40 °C [39].

Model	AIC	$R^{2}$	Para 1	Para 2	Para 3	Para 4	Para 5	Para 6
Model 1	−450.9459	0.9984	3714.2675	281,831.7556	0.0777	0.2927		
Model 2	−447.0584	0.9981	216.7847	3478.4879	0.0512	0.0870		
Model 3	−446.5046	0.9984	398,402.9971	11,697.3221	5353.6713	0.1086	0.0011	0.1089
Model 4	−443.4509	0.9981	537.5333	3142.3824	14.490	0.0801	0.0853	0.1367
Model 5	−446.5763	0.9984	5059.273	9285.55	3622.086	0.0272	0.2397	0.0687
Model 6	−444.0164	0.9982	213,320.028	3756.4477	0.0209	0.1562	0.0830	0.2847
Model 7	−443.0602	0.9981	4822.1409	18,949.6421	93,250.3887	8458.2971	0.8322	0.5246
Model 8	−444.4470	0.9982	9460.9288	755,627.8715	63,810.6481	5904.6320	0.0083	0.1176
Model 9	−445.6424	0.9983	920.9064	5123.0854	0.0112	5693.3887	0.0201	0.1973
Model 10	−446.7083	0.9984	12,546.4112	222,650.4412	5129.6947	45,412.9282	0.1069	0.008
Model 11	−443.5472	0.9981	924,275.4566	0.0231	3708.538	864,974.0201	0.0849	0
Model 12	−442.3771	0.998	763,901.1179	77.7273	409,280.9681	3633.8236	0.3481	0.0874
Model 13	−449.7906	0.9987	3802.2689	106.1188	16,392.3270	31,617.8426	0.1955	0.8579
Model 14	−444.2801	0.9982	1373.8981	7925.7604	40,021.8276	5938.5637	0.292	0.1169
Power-law	−452.8295	0.9984	${7.45\times 10}^{-5}$	${2.1\times 10}^{-4}$	0.1051			
2-term Prony	−382.4545	0.9375	3274.7393	519,860.1096	5657.8364	4.7213	85.0277	
3-term Prony	−444.7925	0.9978	3526.8950	12,641.80	15,423.130	7724.9170	10	100
			1000					
4-term Prony	−446.9092	0.9983	3853.2820	31,784.60	14,762.10	14,073.7410	7974.0824	1
			10	100	1000			

**Table A5:** Optimal fitting parameters of the 14 fractional viscoelastic models, the power-law model, and the Prony series for the compressive creep data of PBX at −4 MPa and 40 °C [39].

Model	AIC	$R^{2}$	Para 1	Para 2	Para 3	Para 4	Para 5	Para 6
Model 1	−632.9381	0.9992	3020.5828	284,582.4783	0.0850	0		
Model 2	−632.1709	0.9992	0.3935	2988.8789	0.6525	0.0843		
Model 3	−637.8925	0.9995	3409.7981	25,907.7757	158,123.1049	0.0663	0.1766	0.0002
Model 4	−627.3614	0.9992	36.5502	1128.0668	1826.3703	0.0186	0.0851	0.0856
Model 5	−642.1545	0.9993	3068.8480	4984.980	67,356.140	0.0633	0.0113	0.3516
Model 6	−636.3960	0.9994	730,620.4340	2890.1018	92.7236	0.3913	0.0801	0.0694
Model 7	−640.8965	0.9995	8875.8666	190.0158	3929.3967	8229.1062	0.1595	0.0809
Model 8	−640.4931	0.9995	4749.5760	34,252.9584	322,903.3525	7591.254	0.7437	0.1466
Model 9	−636.3436	0.9994	0.0103	19,728.9154	0.0756	3496.6525	0.1091	0.0944
Model 10	−637.9497	0.9995	8990.1296	767,586.4616	4372.9914	477,061.7593	0.1102	0.1274
Model 11	−640.5084	0.9995	6551.920	454.3192	4071.8680	12,870.2404	0.1422	0.0606
Model 12	−628.2458	0.9992	0.0124	611,652.3616	3001.6768	105,646.0495	0.0846	0.5344
Model 13	−625.0108	0.9991	2912.6474	0.0878	55,421.5507	16,030.5171	0.9968	0.2081
Model 14	−628.9011	0.9992	17,624.8843	208,466.7441	114,775.6367	3028.2592	0.3285	0.0851
Power-law	−644.0084	0.9995	${1.11\times 10}^{-4}$	${2.42\times 10}^{-4}$	0.11			
3-term Prony	−621.7003	0.9988	2847.1530	10,558.30	12,201.380	6344.6020	10	100
			1000					
4-term Prony	−639.5528	0.9995	3162.0820	21,940	12,808.990	11,084.4130	6503.0036	1
			10	100	1000			

**Table A6:** Optimal fitting parameters of the 14 fractional viscoelastic models, the power-law model, and the Prony series for the compressive creep data of PBX at −5 MPa and 50 °C [39].

Model	AIC	$R^{2}$	Para 1	Para 2	Para 3	Para 4	Para 5	Para 6
Model 1	−393.2690	0.9994	716,387.0310	2140.0558	0.5042	0.0910		
Model 2	−381.1482	0.9986	1724.6198	432.7920	0.0968	0.1021		
Model 3	−378.7898	0.9988	99,985.3337	2331.1266	39,481.4142	0.0048	0.1020	0.0412
Model 4	−377.0171	0.9986	2048.7017	0.0166	108.3436	0.0989	0.2816	0.079
Model 5	−389.0531	0.9993	2141.388	303,228.0	189,801.60	0.0890	0.4143	0.9883
Model 6	−390.1263	0.9994	455.0057	38,139.6923	1686.3339	0.1446	0.7233	0.0775
Model 7	−387.2346	0.9993	36797.1713	1299.2120	86,192.0034	3159.4686	0.1671	0.0518
Model 8	−385.0590	0.9992	47,012.6461	8064.2057	1265.6997	2950.6064	0.1858	0.1197
Model 9	−377.7455	0.9987	52.7636	27,120.3074	1.1052	2272.5669	0.0254	0.1070
Model 10	−382.9867	0.9990	4757.0973	17,030.0111	3756.0060	18,037.3758	0.1425	0.2496
Model 11	−377.3879	0.9986	213,595.2337	0.0106	2177.6142	336.9412	0.0984	0.6067
Model 12	−370.7102	0.9979	151.6239	59,032.0923	2027.3052	452,016.6948	0.1090	0.1063
Model 13	−385.3738	0.9992	2902.4512	591.8831	1419.4994	5965.7615	0.8711	0.2366
Model 14	−385.1609	0.9992	37,904.3095	1290.0287	1009.6874	56,295.5060	0.2432	0.3862
Power-law	−392.8688	0.9992	0.0001	0.0004	0.1213	0		
3-term Prony	−369.2515	0.9971	2057.8530	6086.770	8215.6310	3119.2414	10	100
			1000					
4-term Prony	−400.2626	0.9996	2517.3450	8294.460	8548.7580	6692.9244	3429.9199	1
			10	100	1000			

**Table A7:** Optimal fitting parameters of the 14 fractional viscoelastic models, the power-law model, and the Prony series for the tensile creep data of NEPE propellant at 0.15 MPa [40].

Model	AIC	$R^{2}$	Para 1	Para 2	Para 3	Para 4	Para 5	Para 6
Model 1	−183.7425	0.9625	2.1033	381.9140	0.0393	0.0138		
Model 2	−249.9766	0.9979	1.1649	1.4483	0.0008	0.1819		
Model 3	−178.1034	0.9598	12.0259	2.5071	6233.7111	0	0.0447	0.0004
Model 4	−242.4113	0.9975	1.6048	0.1705	1.1455	0.0187	0.2340	0.3225
Model 5	−235.8586	0.9967	2.4426	5.6815	43.0165	0.0274	0.0089	0.4724
Model 6	−233.8027	0.9964	2.2893	7.9939	1.7292	0.0266	0.0222	0.4542
Model 7	−217.5740	0.9928	2.3095	7.2292	80.5339	30.4587	0.3885	0.0098
Model 8	−240.0415	0.9973	33.7084	3.5576	12.4360	3.2228	0.3619	0.0330
Model 9	−239.9743	0.9973	0.0017	15.479	1.3690	1.8694	0.3486	0.0234
Model 10	−215.8857	0.9922	163,675.1226	44,560.8206	1.3723	2.3354	0.1240	0
Model 11	−245.0710	0.9978	1.4282	6.1381	10.4269	1.6529	0.0281	0.2182
Model 12	−249.4054	0.9982	1.9610	3.3461	12.9339	63.0554	0.9503	0.3812
Model 13	−245.9398	0.9979	5671.7479	1.1452	1.4632	0	0.1787	0.0509
Model 14	−245.7471	0.9979	46.4319	1.2607	1.6487	30.1568	0.1938	0.0439
Power-law	−185.7660	0.9626	0	0.4884	0.0392			
3-term Prony	−203.4257	0.9841	8.1248	2.2355	7.4464	11.2279	10	1000
			100,000					
4-term Prony	−236.2729	0.9965	1.9781	9.1521	18.6345	15.7657	19.0511	100
			1000	10,000	100,000			

**Table A8:** Optimal parameters used for extrapolation for the 3 fractional viscoelastic models, the power-law model, and the 9-term Prony series for the relaxation master curve of PVC/AP propellant [37].

Model	rRMSE	$R^{2}$	Para 1	Para 2	Para 3	Para 4	Para 5	Para 6
Model 5	12.04%	0.9997	1027.1234	12.7111	34.3992	0.0781	0.0328	0.4438
Model 6	5.78%	0.9922	3385.3859	35.5528	10.7414	0.0034	0.4048	0.0237
Model 13	3.45%	0.9915	3591.6887	0	10.7762	35.6670	0.0201	0.4043
Power-law	1.63%	0.7776	6.4828	33.7888	0.2899			
9-term Prony	1.88%	0.9952	6.7762	1759.4788	1802.3137	1199.5601	339.2608	46.6840
			8.9309	2.7817	0.8728	0.4783	$10^{-8}$	$10^{-6}$
			$10^{-4}$	0.01	1	100	10,000	$10^{6}$
			$10^{8}$					

**Table A9:** Optimal parameters used for extrapolation for the 3 fractional viscoelastic models, the power-law model, and the 6-term Prony series for the relaxation master curve of PBX [38].

Model	rRMSE	$R^{2}$	Para 1	Para 2	Para 3	Para 4	Para 5	Para 6
Model 5	2.14%	0.9997	248.6026	34.2305	8.2873	0	0.0779	0.3409
Model 6	8.43%	0.9993	24.8650	734.0214	12.2493	0.1721	0.2274	0
Model 13	2.37%	0.9998	132.8508	7.5066	3.6317	38.8806	0.5542	0.1397
Power-law	8.45%	0.9993	12.1918	21.2299	0.1733			
6-term Prony	13.21%	0.9969	17.7378	134.0490	36.0133	19.9469	8.8925	7.1135
			${3.46\times 10}^{-6}$	${1.26\times 10}^{-4}$	${4.79\times 10}^{-3}$	0.1820	6.9183	$2.63\times 10^{2}$
			10,000					

**Table A10:** Optimal parameters used for extrapolation for the 3 fractional viscoelastic models, the power-law model, and the 4-term Prony series for compressive creep data of PBX at −3 MPa and 40 °C [39].

Model	rRMSE	$R^{2}$	Para 1	Para 2	Para 3	Para 4	Para 5	Para 6
Model 5	4.98%	0.9640	5502.1189	8916.4705	1473.5753	0.0024	0.1638	0.3015
Model 6	0.95%	0.9979	780,752.71	3615.9213	87.7747	0	0.0834	0.1219
Model 13	6.73%	0.9348	3814.872	4425.8546	37,544.39400	0	0.5561	0.0915
Power-law	5.34%	0.9587	${1.73\times 10}^{-4}$	${1.14\times 10}^{-4}$	0.1701			
4-term Prony	14.85%	0.6832	3914.4108	26,111.3620	16,724.4970	15,320.799	3444.1611	1
			10	100	1000			

**Table A11:** Optimal parameters used for extrapolation for the 3 fractional viscoelastic models, the power-law model, and the 4-term Prony series for compressive creep data of PBX at −4 MPa and 40 °C [39].

Model	rRMSE	$R^{2}$	Para 1	Para 2	Para 3	Para 4	Para 5	Para 6
Model 5	1.56%	0.9954	2985.9265	506,527.330	138,527.70	0.0812	0.1220	0.2138
Model 6	1.27%	0.9969	1513.3851	3579.2290	1902.5399	0.1041	0.0208	0.0805
Model 13	7.16%	0.9099	3208.8124	3397.4821	22,607.8730	0	0.5011	0.9942
Power-law	2.45%	0.9587	${1.73\times 10}^{-4}$	${1.14\times 10}^{-4}$	0.1701			
4-term Prony	34.44%	−1.0808	3151.6680	22,902.9040	11,932.9440	161,658.30	1406.9202	1
			10	100	1000			

**Table A12:** Optimal parameters used for extrapolation for the 3 fractional viscoelastic models, the power-law model, and the 4-term Prony series for compressive creep data of PBX at −5 MPa and 50 °C [39].

Model	rRMSE	$R^{2}$	Para 1	Para 2	Para 3	Para 4	Para 5	Para 6
Model 5	2.14%	0.9916	2169.4494	93,128.59	81,055.929	0.0927	0.0753	0.6592
Model 6	1.74%	0.9943	257,703.330	2146.8334	7.7766	0.2075	0.0926	0.1548
Model 13	8.19%	0.8767	2498.2904	2070.4046	9623.7555	892.4337	0.4688	0.8361
Power-law	1.84%	0.9587	${1.01\times 10}^{-6}$	${4.89\times 10}^{-4}$	0.0942			
4-term Prony	3.11%	0.9822	2510.8975	8414.9881	8445.3956	6162.9054	5628.9109	1
			10	100	1000			

**Table A13:** Optimal parameters used for extrapolation for the 3 fractional viscoelastic models, the power-law model, and the 4-term Prony series for creep data of NEPE propellant at 0.15 MPa [40].

Model	rRMSE	$R^{2}$	Para 1	Para 2	Para 3	Para 4	Para 5	Para 6
Model 5	5.03%	0.9158	2.8353	48.6017	6.0325	0	0.9257	0.0804
Model 6	3.62%	0.9563	10.7593	2.4779	4.2406	0.0250	0.0404	0.7737
Model 13	0.56%	0.9979	3.2665	1.6486	4.2508	10.1258	0.1710	0.7184
Power-law	13.45%	0.3488	${1.29\times 10}^{-6}$	0.4290	0.0591			
4-term Prony	0.33%	0.9965	2.0970	7.9937	16.5667	8.1389	86.7225	100
			1000	10,000	100,000			

## Appendix C. Supplementary Evaluation with In-House PBX Creep Data

To provide an additional check beyond the published datasets, tensile creep data for a TATB-based PBX were obtained at 20 °C under a stress level of 5 MPa using an electronic universal testing machine. The creep strain was measured using an extensometer. The specimens were fabricated by hot isostatic pressing and machined into dog-bone-shaped specimens according to GJB 772A-1997 [41]. The specimens were first loaded to 5 MPa under displacement control at 0.5 mm/min, after which the stress was held constant for the creep test. The obtained creep data covered a time span of 0–20,000 s. The fitting performance of the fractional Zener model, Model 13, the power-law model, and the 3-term and 4-term Prony series models was further evaluated, as shown in Figure A1 and Table A14.

**Table A14:** Coefficients of determination and Akaike information criterion corresponding to the compared models for the in-house TATB-based PBX creep data.

Model	FZM	Model 13	Power Law	3-Term Prony	4-Term Prony
$R^{2}$	0.9906	0.9974	0.9978	0.9782	0.9968
AIC	−1034.6818	−1083.4135	−1074.6890	−1007.5034	−1076.8689

All models except the 3-term Prony series achieved satisfactory fitting performance for the experimentally obtained tensile creep data of the TATB-based PBX. The fractional Zener model, Model 13, the power-law model, and the 4-term Prony series all gave coefficients of determination higher than 0.99. Among them, the power-law model yielded the highest $R^{2}$ value ($R^{2}=0.9978$), followed closely by Model 13 ($R^{2}=0.9974$) and the 4-term Prony series ($R^{2}=0.9968$). When model complexity was further considered using AIC, Model 13 exhibited the lowest AIC value (AIC=−1083.4135), indicating the most favorable balance between fitting accuracy and parameter complexity for this dataset. The residual plots in Figure A2 further support this result. The residuals of the fractional Zener model and Model 13 were distributed relatively uniformly around zero over the investigated time range, without obvious systematic deviation, indicating that these models captured both early-stage and long-time creep responses of the TATB-based PBX.

These supplementary results based on independently measured TATB-based PBX creep data are consistent with the main trends obtained from the published datasets. In particular, Model 13 and the fractional Zener model provided accurate creep descriptions with a limited number of parameters, while maintaining favorable parameter efficiency and rheological interpretability. Therefore, the additional dataset supports the applicability of the selected fractional models to PBX creep analysis. Nevertheless, this supplementary assessment should be regarded as an additional consistency check rather than as complete independent validation over all loading conditions.

As shown in Figure A3 and Table A15, when the first 10% time span of the experimentally obtained TATB-based PBX creep data was used for parameter identification, the fractional Zener model, Model 13, and the power-law model all exhibited satisfactory extrapolation performance, whereas the Prony series models showed much less stable extrapolation behavior.

**Table A15:** Extrapolation performance of the compared models for the in-house TATB-based PBX creep data.

Model	FZM	Model 13	Power Law	3-Term Prony	4-Term Prony
$R^{2}$	0.9849	0.9734	0.9848	−0.7987	0.6046
rRMSE	0.76%	1.03%	0.76%	8.76%	4.10%

Overall, the selected low-order fractional viscoelastic models, especially the fractional Zener model and Model 13, showed acceptable extrapolation performance with a limited number of parameters. In particular, the fractional Zener model and Model 13 achieved low rRMSE values of 0.76% and 1.03%, respectively, indicating that both fractional models can provide reliable extrapolation for PBX creep data characterized by moderate steady-state creep rates.

## Appendix D. Stability of Parameter Identification

To further evaluate the stability of the parameter identification procedure, 30 independent optimization runs were performed for the same model and dataset using different random streams. The identified parameters from these runs were statistically analyzed, and their distributions are shown in Figure A4. Specifically, $E_{1}$ was mainly distributed within 3400–3900 MPa and $E_{2}$ within 0–8 MPa, while $\eta_{1}$ and $\eta_{2}$ were mainly distributed within 0–11 $MPa\cdot s^{\alpha }$ and 29–41 $MPa\cdot s^{\beta }$, respectively. The fractional order α fluctuated around 0.4, whereas β was distributed within 0–0.5. Although moderate variations were observed for some parameters, the overall distributions remained bound and showed no significant divergence among different runs. This indicates that the optimization process converged toward a stable parameter region rather than randomly scattered local solutions.

The consistently high $R^{2}$ values in Figure A5a further indicate that Model 13 maintained stable descriptive accuracy under different random initializations. The convergence history of the third independent run, shown in Figure A5b, exhibits a rapid decrease in the objective function during the early iterations, followed by a stable plateau. This indicates effective convergence of the optimization process.

The fitted curves obtained from the 30 independent runs almost overlapped with each other and closely followed the PVC/AP relaxation master curve, as shown in Figure A5c. Moreover, the corresponding extrapolated curves over the approximately one-order-of-magnitude longer time window also showed negligible differences, as shown in Figure A5d. These results suggest that moderate variations in individual parameters had limited influence on the macroscopic relaxation response and its early-stage data extrapolation behavior.

To examine possible parameter coupling, the correlation coefficients among the identified parameters ($E_{1},\;E_{2},\;\eta_{1},\;\eta_{2},\;\alpha ,\;\beta$) from the 30 independent runs were calculated, as shown in Matrix (A1). The correlation matrix indicates that some parameters were moderately or strongly correlated, suggesting that the identified parameter set may not be strictly unique. This behavior is common in multi-parameter fractional viscoelastic models, where different parameter combinations can produce similar macroscopic responses. Nevertheless, the fitted relaxation curves and the corresponding extrapolated curves remained nearly unchanged among the 30 independent runs. This indicates that, although parameter correlation exists, the response-level identification result is stable for the representative case considered.

(A1) $$C=\left[\begin{array}{cccccc} 1 & 0.3260 & 0.9733 & -0.8450 & -0.3409 & -0.0900\\ 0.3260 & 1 & 0.1890 & -0.7592 & -0.0085 & -0.1356\\ 0.9733 & 0.1890 & 1 & -0.7814 & -0.3617 & -0.1356\\ -0.8450 & -0.7592 & -0.7814 & 1 & 0.2425 & -0.1640\\ -0.3409 & -0.0085 & -0.3617 & 0.2425 & 1 & 0.4217\\ -0.0900 & -0.1356 & -0.1356 & -0.1640 & 0.4217 & 1 \end{array}\right],$$

Overall, the repeated identification results and parameter correlation analysis support the stability of the combined Talbot–Gray Wolf parameter identification procedure at the response level for the representative case considered. Although individual parameters may exhibit correlation and are not necessarily uniquely determined, the fitted and extrapolated curves remain nearly unchanged among independent runs. These results support response-level stability of the identification procedure for the representative case, although they also indicate that strict parameter uniqueness cannot be guaranteed.
